# Dual Role of Extracellular Vesicles in Sepsis-Associated Kidney and Lung Injury

**DOI:** 10.3390/biomedicines10102448

**Published:** 2022-09-30

**Authors:** Marco Quaglia, Vito Fanelli, Guido Merlotti, Andrea Costamagna, Maria Chiara Deregibus, Marita Marengo, Eleonora Balzani, Luca Brazzi, Giovanni Camussi, Vincenzo Cantaluppi

**Affiliations:** 1 Nephrology and Kidney Transplantation Unit, Department of Translational Medicine, University of Piemonte Orientale (UPO), 28100 Novara, Italy; 2 Department of Anaesthesia, Critical Care and Emergency, Città della Salute e della Scienza Hospital, University of Torino, 10126 Torino, Italy; 3 Department of Medical Sciences, University of Torino, 10126 Torino, Italy; 4 Nephrology and Dialysis Unit, ASL CN1, 12038 Savigliano, Italy

**Keywords:** sepsis, acute kidney injury, acute respiratory distress syndrome, extracellular vesicles, mesenchymal stromal cell, regenerative medicine

## Abstract

Extracellular vesicles form a complex intercellular communication network, shuttling a variety of proteins, lipids, and nucleic acids, including regulatory RNAs, such as microRNAs. Transfer of these molecules to target cells allows for the modulation of sets of genes and mediates multiple paracrine and endocrine actions. EVs exert broad pro-inflammatory, pro-oxidant, and pro-apoptotic effects in sepsis, mediating microvascular dysfunction and multiple organ damage. This deleterious role is well documented in sepsis-associated acute kidney injury and acute respiratory distress syndrome. On the other hand, protective effects of stem cell-derived extracellular vesicles have been reported in experimental models of sepsis. Stem cell-derived extracellular vesicles recapitulate beneficial cytoprotective, regenerative, and immunomodulatory properties of parental cells and have shown therapeutic effects in experimental models of sepsis with kidney and lung involvement. Extracellular vesicles are also likely to play a role in deranged kidney-lung crosstalk, a hallmark of sepsis, and may be key to a better understanding of shared mechanisms underlying multiple organ dysfunction. In this review, we analyze the state-of-the-art knowledge on the dual role of EVs in sepsis-associated kidney/lung injury and repair. PubMed library was searched from inception to July 2022, using a combination of medical subject headings (MeSH) and keywords related to EVs, sepsis, acute kidney injury (AKI), acute lung injury (ALI), and acute respiratory distress syndrome (ARDS). Key findings are summarized into two sections on detrimental and beneficial mechanisms of actions of EVs in kidney and lung injury, respectively. The role of EVs in kidney-lung crosstalk is then outlined. Efforts to expand knowledge on EVs may pave the way to employ them as prognostic biomarkers or therapeutic targets to prevent or reduce organ damage in sepsis.

## 1. Introduction

### 1.1. General Features and Biological Activities of Extracellular Vesicles

Extracellular vesicles (EVs) are microparticles that are created by cytosol, which are surrounded with a bilayer membrane with protein and lipid composition and released by cells into the extracellular environment. Vesiculation is a well-maintained process throughout evolution, which is present in plants, prokaryotes, and eukaryotes [1]. EVs have been described in almost all fluids of the human body in physiological or pathological circumstances. Since the EV cargo include selectively sorted molecules, such as lipids, proteins, and nucleic acids, the transfer of these molecules among cells by EVs is feasible and represents a key component of intercellular communication. This review discusses the current understanding of the role of EVs in kidney and lung acute dysfunction in relation to sepsis. Moreover, it provides the newest evidence on the role of EVs from stem cells as a novel therapeutic target in this complex clinical scenario.

EVs consist of heterogeneous populations of vesicles of different size, morphology, and composition originating from various cell compartments [2]. The membrane vesicles with size between 30 and 100 nm derived from the cell multivesicular bodies are called exosomes. They bud from endosomal membranes of multivesicular bodies, fuse with the surface of the cell, and are released into the extracellular space [3]. Ectosomes consist of various populations of vesicles originated by plasma membrane shedding into the extracellular environment [4,5]. Ectosomes, also called microvesicles or microparticles, comprise vesicles of size between 50 and 250 nm that are released from healthy cells and vesicles with size up to 1 µm, including the pre-apoptotic vesicles [4,5]. Vesicles derived from cells undergoing apoptosis, with size between 1 and 5 µm, and containing nuclear fragments are named apoptotic bodies [6]. The biogenesis of the diverse classes of EVs has not been completely explained. It has been suggested that some elements of the endosomal sorting complex required for transport (ESCRT) machinery and proteins implicated in multivesicular body sorting, such as apoptosis-linked gene 2-interacting protein X (ALIX), tumor susceptibility gene 101 (TSG101), and vacuolar protein sorting-associated 4 (VPS4) are implicated not only in the biogenesis of exosomes, but also of ectosomes. In addition, a study showed another mechanism of exosome biogenesis independent from ESCRT machinery [7].

The EV cargo is complex and comprises a variety of biological active proteins, lipids, and nucleic acids. The cargo composition reflects the type and condition of the cell of origin and the physiological or pathological state. In general, EVs share some proteins normally expressed by all EVs similar to those associated with their formation from multivesicular bodies (TSG101 and ALIX), proteins related to membrane transport and fusion (GTPases, annexins, and flotillin), tetraspanins (CD9, CD63, CD81), major histocompatibility complexes I and II, growth factors and receptors, signaling and cell adhesion molecules, transcription factors and cytokines. Moreover, EVs express molecules characteristic of cells from which they derive and the presence of the molecular signature of the cell of origin has been exploited for diagnostic purposes [8]. In addition, it has been demonstrated that nucleic acids are a relevant component of the EV cargo and their transfer to target cells may induce phenotypic and functional changes [9,10]. Cargo sharing between different cells represents a mechanism of cell-to-cell communication involved in physiological and pathological processes [11]. The biological activities of EVs are related to the transfer of transcripts that may target pathways in the recipient cells and may include not only regulatory RNAs, such as mRNAs, microRNAs, and long-noncoding RNAs, but also growth factors [12]. Moreover, EVs may be exploited for therapeutic purposes using engineered EVs [13] or native EVs, such as those derived from stem/progenitor cells [14,15,16]. In the last years, several studies demonstrated that most of the biological activities of EVs are correlated with the horizontal transfer of their RNA cargo to target cells [9,11]. In particular, the role of specific mRNAs and miRNAs carried by stem cell-derived EVs to injured tissues (mostly in experimental AKI models) was confirmed [9,10,17].

Recent studies showed that EVs are key regulators of immune system in sepsis, which is released by a variety of both immune and non-immune cells and are involved in mechanisms of sepsis-induced multi-organ failure, including kidney and lung damage [18].

### 1.2. Sepsis and Multi-Organ Failure—New Potential Mechanisms

Sepsis is characterized by an abnormal immune response secondary to bacterial, viral or fungal infection, causing multi-organ dysfunction syndrome (MODS) and death [19]. Between 1997 and 2017, sepsis was the leading cause of mortality in intensive care unit (ICU) patients [20]. Mortality ranges from 25–30% to 50% in patients with septic shock [21,22], but it can reach 90% in MODS with four or more dysfunctional organ systems [23].

Antibiotic therapy, fluid resuscitation, and vasopressor treatment are the mainstay of therapy, whereas renal replacement therapy (RRT) and mechanical ventilation may be required for concomitant renal or respiratory failure.

The mechanisms underlying the evolution from sepsis to MODS are not yet fully elucidated. Disruption of redox homeostasis resulting in oxidative stress, combined with over-inflammation, is thought to cause mitochondrial and microvascular dysfunction [23,24]. Hypotension due to peripheral vasodilation, a hallmark of sepsis, may be poorly responsive to norepinephrine [25] and result in tissue hypoxia and generalized mitochondrial dysfunction [24].

Kidney and lung are often involved when MODS develops; the etiopathogenetic peculiarities of sepsis-associated acute kidney injury (s-AKI) and acute respiratory distress syndrome (ARDS) will be separately analyzed in the next paragraphs to discuss the role of EVs as both detrimental mediators of organ damage and potential therapy in this challenging setting.

## 2. Sepsis-Associated Acute Kidney Injury

According to KDIGO, AKI is defined as an increase in serum creatinine by 0.3 mg/dL within 48 h or as an increase in serum creatinine to ≥1.5 times from baseline, known or presumed to have occurred within the previous 7 days or a urine volume <0.5 mL/kg/hour for 6 h [26]. AKI is common in septic patients and associated with increased morbidity and mortality [27]. In a recent study of 1243 patients with septic shock, 69.1% developed AKI by KDIGO criteria and the development of s-AKI is associated with a 5-fold increase in 60-day mortality [28]. Risk factors for s-AKI include advanced age, CKD, cardiovascular disease, diabetes and heart failure. On the other hand, AKI per se can increase the risk of sepsis, its severity, and related adverse outcomes [29].

An important point is that the dysfunction of other organs is one of the main causes of poor outcomes from AKI: The inflammatory response following AKI, due to the loss of tubular function, has been shown to induce early and late cardiovascular, brain, lung, liver, and immune dysfunction even in the absence of progression toward CKD [30,31,32].

In the last years, s-AKI has been shown to be not only a consequence of ischemic damage due to hypoperfusion, but also of pathogenic mechanisms that are more toxic and immunologic in nature. These include microvascular damage and intrarenal redistribution of renal blood flow, activation of immune cells and complement system with massive release of inflammatory molecules causing renal tubular epithelial cell (RTECs) dysfunction and damage (cell cycle arrest, dedifferentiation, activation of autophagy and mitophagy, loss of polarity, apoptosis) [33].

In particular, the EV-mediated horizontal transfer of different RNA subtypes to injured microvascular endothelial cells (ECs) and RTECs may play a pivotal pathogenetic role in these processes. Our group has previously demonstrated that plasma of patients with severe sepsis and septic shock contains circulating pro-apoptotic and pro-inflammatory factors responsible for a direct damage of human glomerular and tubular epithelial cells [34]. At least part of these septic plasma-induced functional and lethal alterations might be ascribed to the presence of circulating EVs, which can be considered as tangible damage associated molecular patterns (DAMPs) molecules. In the condition of increased glomerular permeability, EVs can reach tubular lumen and exert their detrimental activities on ECs located in peritubular capillaries, thus contributing to microvascular derangement, thrombo-inflammation, and consequent bioenergetic alterations of RTECs [35].

On the other hand, over the last years regenerative medicine has acquired a key role in different experimental models of acute tissue damage, including AKI [36]. Of note, the protective mechanisms exerted by stem cells (SCs) on kidney injury are mainly ascribed to the release of paracrine mediators, such as growth factors and EVs. Several studies in experimental AKI models showed a protective effect of hematopoietic and mesenchymal stem cells (MSC) released from bone marrow and other sources, including adipose tissue, cord blood, placenta, etc. Similar results were obtained using progenitor cells of mesenchymal origin isolated from the kidney and committed to endothelial and tubular epithelial cell differentiation or directly employing kidney-derived EVs isolated from urine [37].

The pathogenetic role of EVs and the therapeutic potential of SC-derived EVs in s-AKI will be analyzed in Section 2.1 and Section 2.2, respectively.

### 2.1. Role of EVs as Mediator of Renal Damage in s-AKI

As previously highlighted, both detrimental and beneficial effects of EVs in s-AKI can be mainly ascribed to the transfer of RNA subtypes to target cells. In this section, we will analyze the detrimental role of EVs in s-AKI, focusing on miRNAs which appear to be involved in kidney damage.

Although there is limited evidence of a specific role of EVs as mediator of renal damage in s-AKI, it is plausible that many EV-related biological activities described in ischemic and toxic AKI may also apply to this setting. EVs can indeed modulate key intra-renal mechanisms involved in s-AKI, such as microvascular dysfunction, thrombo-inflammation, hypoxic and/or oxidant stress, altered crosstalk between RTECs and immune cells, cytokine-driven tubular damage.

A role of circulating EVs in these mechanisms of tissue injury may at least in part explain some findings observed in experimental AKI models and corroborated by clinical observations. In particular, the dissociation between renal function and blood flow in s-AKI has been clearly demonstrated. Indeed, AKI develops in the presence of a normal or even increased renal blood flow, suggesting that mechanisms other than hypoperfusion should sustain tissue damage, thus highlighting the role of circulating mediators. s-AKI seems to be different from other forms of AKI due to an increased mortality rate and a propensity to progression toward CKD, through mechanisms of accelerated kidney senescence [38,39].

On this basis, we will herein focus on the role of EVs as mediators of some of these peculiar aspects of renal damage in s-AKI [35,40].

#### 2.1.1. EVs and Microvascular Dysfunction

As previously described, s-AKI is characterized by heterogeneous zones of sluggish blood flow, which are associated with areas of RTECs oxidative stress, despite normal or even increased renal blood flow [40].

This microvascular derangement recognizes multiple contributing factors, such as endothelial dysfunction and damage (shedding of glycocalyx) and consequent capillary leak, leukocyte adhesion, activation of coagulation, and thrombo-inflammation [41].

EVs can be involved in several aspects of this pathophysiological process, as demonstrated for other inflammatory renal diseases [42] as well as for lung during sepsis [43].

Increased circulating levels of EVs in sepsis (mainly derived from PLTs and ECs) can directly affect the endothelial production of nitric oxide, prostacyclin, and inflammatory cytokines by modulating the expression of related specific genes (nitric oxide synthases, cyclooxygenase-2, and nuclear factor-κB, respectively). This leads to impaired vasorelaxation, increased oxidative and nitrosative stress (described in more detail in Section 2.1.2), rolling and adhesion of leukocytes and PLTs to the endothelium [44]. Consequently, hemodynamic alterations develop in systemic and renal microcirculation, with sluggish or intermittent blood flow [45].

In addition to these effects, EVs have strong pro-thrombotic and pro-coagulant properties in sepsis and are involved in pathogenesis of diffuse intravascular coagulation (DIC) through several mechanisms. EVs released by PLTs, but also ECs and monocytes, can expose phosphatidylserine on their surface, catalyzing the interaction between coagulation factors and tissue factor (TF), a major trigger of the extrinsic pathway of the coagulation cascade. EVs with this pro-coagulant phenotype can lead to thrombi formation in the microcirculation of different organs, including the kidney [46,47].

Of interest, PLT-derived EVs appear to be elevated in sepsis patient and to inversely correlate with renal function [48].

All these EV-mediated effects are likely to contribute to detrimental hemodynamic alterations of s-AKI, leading to redistribution of intra-renal perfusion and medullary hypoxia [49].

Similar alterations of endothelial function have been recently observed in COVID-19, another disease characterized by significant lung-kidney interactions similar to bacterial sepsis. SARS-CoV-2 can enter ACE-2 expressing ECs in glomerular and peritubular capillaries and trigger inflammation (e.g., release of IL-1 and IL-6), coagulation and complement cascade [50]. In this setting, several PAMPs and DAMPs may cooperate to induce endothelial dysfunction in the lung and in the kidney with similar mechanisms [51]. It has been shown that the spike protein of SARS-CoV-2 is able to modulate LPS aggregation, thus enhancing its pro-inflammatory properties [52]. Of interest, platelet-derived EV count is higher in COVID-19 patients and represents an independent predictor of outcome. Moreover, proteomic analysis of EVs from plasma of COVID-19 patients identified several molecules involved in immune response, inflammation, activation of coagulation and complement cascade, in addition to the presence of SARS-CoV-2 RNA; this suggests that EVs may be used by the virus as an endocytosis route to spread infection and represent an important mediator of microvascular damage [53,54].

#### 2.1.2. EVs and Oxidative Stress

Both endothelial and PLT-derived EVs can affect redox reactions as they carry nicotinamide adenine dinucleotide phosphate (NADPH) oxidase (NOX) subunits catalyzing production of superoxide anion and other ROS in sepsis [55].

EV-induced oxidative stress can injure not only EC, but also RTEC during sepsis. PLT-derived EVs can induce copper/zinc superoxide dismutase in kidneys and other organs to a higher extent than observed in healthy state [44].

Endothelial EVs may exert anti-angiogenic effects through similar mechanisms amplifying ROS release and oxidative stress [56,57].

Of interest, angiotensin 2 can directly induce the release of endothelial EVs in sepsis through a pathway, including NADPH oxidase, whereas EVs themselves can in turn stimulate ROS release by ECs, creating a feedforward mechanism of EV-mediated vascular damage [58].

Moreover, urinary EVs (uEVs) have been shown to carry a specific miRNA profile which correlates with ischemia-reperfusion injury of s-AKI [59].

Considered together, these elements suggest a biological role of RNA subtypes carried by circulating EVs in the induction of s-AKI. In particular, the absence of overt necrosis or apoptosis has led to the hypothesis that RTECs may deploy defense mechanisms to survive the insult, suggesting that the defense against infection also depends on the capacity of cells and tissues to limit damage [35,60]. In this setting, RTECs are immunologically active cells, capable of presenting antigens, but also dedicated to the clearance of inflammatory mediators [61].

During AKI, PAMPs and DAMPs, including EVs, may also contribute to mitochondrial dysfunction and delay in the repair of injured tissues [62]. Indeed, mitochondria generate ATP to support tubular function, including antioxidant responses, autophagy, and mitochondrial quality control. Nicotinamide adenine dinucleotide (NAD+) is essential for the preservation of tubular health and integrity mainly through the expression of PGC1-alpha, a key-protein for mitochondrial biogenesis [63].

#### 2.1.3. EVs and Immune Dysfunction

In addition to direct effects on kidney resident cells, such as ECs and TECs, circulating plasma EVs may also exert some indirect effects in s-AKI through modulation of the immune response. EVs can exert a dual effect: on the one hand, they can promote inflammation, whereas, on the other hand, they can mediate immunosuppression in sepsis [18].

The first aspect appears to be relevant to kidney damage. Chemotactic and pro-inflammatory effects can be determined by molecules carried by circulating PMN-derived EVs (such as monocyte chemoattractant protein 1 and galectin-3), PLT-derived EVs (RANTES and P-selectin), and EC-derived EVs (IL-8) [42,64].

RTECs are the most represented cell type within the kidney and play a key role in s-AKI by releasing EVs which can activate neighboring macrophages, mediating a crosstalk between these two cell types. Of note, macrophages polarization is an important aspect in pathogenesis of sepsis. M1-macrophages generate a pro-inflammatory milieu which favors bacteria phagocytosis but related cytokines correlate with mortality in severe sepsis. On the other hand, M2 macrophages are predominant in the reparative phase and in healthy kidneys [65].

At kidney level, RTEC-derived EVs can polarize macrophages toward M1 phenotype, inducing them in turn to release EVs which promote tubular injury and interstitial inflammation [66].

Specific miRNAs, such as miR-19b-3p, are highly expressed in RTEC-derived EVs and mediate macrophage activation through targeting NF-κB/suppressor of cytokine signaling-1 (SOCS-1) in an LPS-induced AKI mouse model [67]. The miR-19b-3p/SOCS-1 axis appears to play a critical pathologic role in tubulointerstitial inflammation, as confirmed by the correlation of high levels of miR-19b-3p-carrying EVs with severity of this pathological aspect also in diabetic nephropathy [68].

In a similar way, miR-23 transferred by hypoxic RTEC-derived EVs can promote M1 polarization of kidney resident macrophages [69].

Increased release of EVs that transfer CCL2 mRNA from BSA-treated RTECs to macrophages is another mechanism leading to macrophage migration and tubulointerstitial inflammation [70].

On the other hand, M1 macrophages regulate AKI by secreting EVs enriched in miR-93-5p, which directly influence pyroptosis in RTECs, completing this crosstalk between RTECs and macrophages [71,72].

Of note, EVs also represent an intra-nephron, paracrine communication system connecting tubular cells of different segments between them and with interstitial cells [73].

After a systemic injury, EVs from proximal RTEC can mediate interaction with infiltrating macrophages and fibroblasts within the interstitial compartment, promoting inflammation and evolution toward interstitial fibrosis. This mechanism may contribute to AKI-CKD transition [74].

On the other hand, proximal RTEC-released EVs after stimulation with fenoldopam can reach distal tubular cells, reduce their ROS production and modulate expression of solute-transporting proteins, suggesting a regulatory role within repair responses [66].

**Figure 1:**
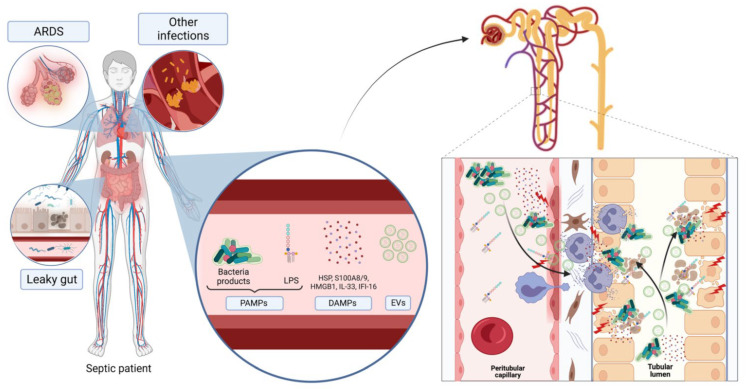
Kidney injury mediators in sepsis (Created with Biorender.com, accessed on 28 August 2022).

In conclusion, EVs mediate major recognized mechanisms of s-AKI damage (microvascular and immune dysfunction, oxidative stress) through a variety of actions involving ECs, RTECs, macrophages and other immune cells (Figure 1). A better characterization of EV content (especially miRNAs) could help improve our knowledge of the pathophysiology of s-AKI and potentially provide new therapeutic targets.

### 2.2. Role of Stem Cell-Derived Extracellular Vesicles as a Potential Therapeutic Tool in s-AKI

Mounting evidence indicates a potential therapeutic role of EVs derived from MSC and other stem cell types in pre-clinical models of ischemic and toxic AKI, whereas there is still a paucity of data on the role of EVs in the specific setting of s-AKI repair.

In the former models, EVs can shuttle miRNAs and other genetic material into injured RTECs and ECs and epigenetically re-program them. This leads to activation of multiple signaling pathways and confers beneficial effects, which can be categorized within three main areas [75,76,77]:

- Renal protection: inhibition of oxidative stress, apoptosis, and fibrogenesis; promotion of autophagy [78].
- Renal regeneration: stimulation of cell proliferation, migration, tubular dedifferentiation, angiogenesis [79].
- Immunomodulation: anti-inflammatory and immunosuppressive effects, through induction of M2 macrophages and T-regulatory cells (Treg) [80] and modulation of NK cells [36,81].

The combination of these effects can promote repair of injured RTECs. Of note, pre-treatment with RNAase consistently abolished them, indicating a crucial role of mRNAs and/or miRNAs transfer [82].

Ferguson et al. actually identified 23 top-miRNAs which seem to mediate their main actions, targeting 5481 genes [83], and miRNA repertoire carried by SC-derived EVs employed as AKI therapy has been published [84].

Consistent with this background, initial evidence suggests that EVs from different cell types can have beneficial effects in s-AKI and pivotal miRNAs are being identified, as described in Table 1.

For example, endothelial progenitor cell (EPC)-derived EVs proved to alleviate s-AKI modulating miR-21-5p/runt-related transcription factor 1 (RUNX1) axis in a cecal ligation and puncture (CLP) rat model. Elevation of miR-21-5p improved renal function and pathological lesions, reducing tissue apoptosis and oxidative stress. Moreover, EPC-derived EVs containing miR-21-5p modulated syndecan-1 and heparinase-1, both markers of endothelial glycocalyx damage [85].

Furthermore, EPC-derived EVs carrying miR-93-5p conferred endothelial protection in an LPS-induced mouse model of s-AKI with MOD [86] and blunted LPS-induced HK-2 cell injury in another model [87]. This miRNA physiologically downregulates thioredoxin-interacting protein (TXNIP), a physiological inhibitor of thioredoxin antioxidant activity, which is pathologically enhanced in diabetes and cardiovascular disease and involved in inflammation [93].

A recent study confirmed that exosomal miR-93-5p released from macrophages, in which it is differentially expressed, directly regulated TXNIP and thus influenced pyroptosis in RTECs [71].

Many long non-coding RNAs have been found to play crucial roles in s-AKI by modulating miRNAs. For example, “nuclear-enriched abundant transcript 1” (NEAT-1), which is upregulated in LPS-induced human tubule epithelial HK-2 cells, results in miR-93-5p inhibition and consequently it aggravates LPS-induced injury in HK-2 cells by modulating miR-93-5p/TXNIP axis [94].

In addition, miR-22-3p is significantly downregulated in a rat model of s-AKI in vivo and LPS-induced sepsis model in HK-2 cells in vitro. Moreover, miR-22-3p can suppress inflammatory response and apoptosis downregulating HMGB1, p-p65, TLR4, and pro-inflammatory cytokines (IL-1β, IL-6, TNF-α), both in vivo and in vitro. Furthermore, it can repress phosphatase and tensin homologue (PTEN), a protein involved in mitophagy regulation, thus playing a protective role in s-AKI [88].

Human MSC-EVs significantly improved renal function, morphological damage, and even 72-h survival (from 28% to 45%) in a sepsis mouse model through CLP. Of interest, MSC-EVs increased expression of miR-146b in kidney tissue and consequently reduced interleukin-1 receptor-associated kinase (IRAK1) level and NF-κB activity, resulting in blunted inflammatory response [89].

Adipose-tissue derived-EVs (AT-EVs) have also proved effective in a CLP mouse model, activating SIRT1 signaling pathway and improving renal function and survival [90].

EVs derived from mice pre-treated with remote ischemic preconditioning, elicited by brief periods of IRI in femoral arteries, appear to protect against s-AKI through miR-21, which integrate into RTECs and target the downstream PDCD4/NF-κB and PTEN/AKT pathway [91].

MSC-EVs attenuated mtDNA damage and inflammation after AKI and this effect was partially dependent on the mitochondrial transcription factor A (TFAM) pathway. Moreover, loss of TFAM led to downregulation of multiple anti-inflammatory miRNAs and proteins in MSC-EVs [92].

## 3. Role of Extracellular Vesicles in Sepsis-Associated ARDS

ARDS is defined as the presence of respiratory failure in a patient with bilateral opacities at chest imaging, within 1 week of a known clinical insult or new or worsening respiratory symptoms. Cardiac failure or fluid overload must be excluded as the leading cause of respiratory impairment and lung edema. ARDS is classified according to hypoxia severity into a mild, moderate or severe form if the ratio between the partial pressure of arterial oxygen and the fraction of inspired oxygen (PaO_2_/FiO_2_) is among 200 and 300 mmHg, among 100 and 200 mmHg or below 100 mmHg, respectively [95]. ARDS may be triggered by a wide range of noxious stimuli, with sepsis as the most frequent etiology, thus in fact contributing to the worst outcomes [96,97,98]. In fact, data from the LUNGSAFE study, an international, multicenter, prospective cohort study conducted in 2014 on a sample of 459 ICUs and enrolling more than 29,000 ICU patients, showed that among 4499 patients with hypoxemic respiratory failure, about 3000 (10%) subjects satisfied the ARDS criteria according to the Berlin definition of ARDS. Several risk factors for ARDS development were recognized and pneumonia and extrapulmonary sepsis accounted for 60% and 16% of cases, respectively [96]. Data from the 2017 Global Burden of Diseases, Injuries, and Risk Factors Study, estimating the incidence of sepsis and sepsis-related deaths in every year from 1990 to 2017 across 195 countries, showed that lower respiratory tract infections were the second most common underlying cause of sepsis in 2017 and the most common underlying cause of sepsis-related deaths in every year from 1990 to 2017 [20]. Therefore, it can be deduced that sepsis in general represents the leading cause of ARDS in more than 75% of cases. In addition, the worldwide emergence in the last two decades of pandemics driven by viral pathogens—namely H1N1 Influenza A after 2009 and SARS-CoV-2 after 2019—determined an increase in terms of pneumonia-related ARDS incidence during epidemic and pandemic waves [97,99,100].

ARDS is characterized by alveolar epithelial and endothelial barrier damage [101,102], leading to the accumulation of protein-rich alveolar edema, impairment of the surfactant homeostasis, and dysregulation of the active epithelial fluid transport system, which is implied in edema reabsorption and resolution of the respiratory failure [103]. The activation of macrophage, neutrophil, and monocyte leads to a sustained inflammation, perpetuating tissue injury. Depending on the intensity of noxious stimuli and of dysregulated inflammatory response, pulmonary fibrosis may occur in the late phase of ARDS, with poor patient outcome [101,102].

Treatment relies basically on supportive therapy, focusing on protective ventilatory strategies aimed at avoiding further iatrogenic lung damage (i.e., ventilator-induced-lung injury) and conservative fluid balance. Rescue therapies, such as prone positioning, neuromuscular blockade, and extra-corporeal membrane oxygenator (ECMO) are reserved for refractory or worsening moderate-to-severe cases. Presently, no pharmacologic therapy showed benefits in terms of survival. The administration of mesenchymal stem cells and/or their secretome with the aim of helping to restore the injured lung tissue is fueling significant expectation [102].

The pathogenetic role of EVs and the therapeutic potential of SC-derived EVs in ARDS will be analyzed in Section 3.1 and Section 3.2, respectively.

### 3.1. Role of EVs as Mediators of Lung Damage in Sepsis-Associated ARDS

Different types of lung cells release EVs involved in tissue damage in the course of sepsis-associated ARDS.

Alveolar macrophages (AMs) are the main source of innate immune system activation against invading respiratory pathogens or pro-inflammatory sterile insults to the lungs. They greatly contribute to acute lung injury (ALI) through overwhelming inflammation and can be activated by EVs released from damaged cells of lung epithelial/endothelial barrier. Furthermore, they secrete EVs which further aggravate lung injury. In a mouse model of hyperoxia-induced ALI, isolated AMs treated with EVs derived from lung epithelial cells released pro-inflammatory mediators, such as IL-6, TNF-α, and MIP-2 [104]. Interestingly, time course of EVs released from different alveolar cells has been described. One hour after LPS administration, AMs were the main source of EVs, followed 3 h later by a prevailing production of EVs from epithelial cells and neutrophils. AM-derived EVs induced ICAM-1 expression in epithelial cells in vitro, as well as ALI after intratracheal administration in mice. These data suggest the potential role of pro-inflammatory AM-derived EVs cargo in initiating the early phases of lung injury [105].

EV profile also changes according to the nature of the lung insult, suggesting that EVs may serve as a biomarker of high-permeability lung edema. Lung exposure to sterile (i.e., oxidative stress and acid aspiration) and infectious stimuli (i.e., LPS and Gram negative bacteria) induced the release of EVs from lung alveolar type-I epithelial cells or AMs, respectively. Regardless of their origin, both types of EVs contributed to in vivo macrophage recruitment, leading to overwhelming lung inflammation [106]. However, EVs released in high-permeability lung oedema are different from those in hydrostatic pulmonary oedema. In fact, EVs were more abundant in BALF of ARDS patients compared with patients with hydrostatic oedema and had greater pro-coagulant activity, mediated by a higher expression of TF [107]. Compared with septic patients without lung involvement, patients with sepsis-related ARDS had higher number of CD14+/CD81+ monocyte-derived EVs in BALF and this signature was associated with dismal outcome [108].

Furthermore, patients at early and late phases of ARDS can display a characteristic EV profile that differs from patients undergoing mechanical ventilation. EVs containing secretory phospholipase A2 (sPLA2) were present in BALF of patients with early ARDS, together with higher expression of its specific mRNA, i.e., PLA2G2A and were associated with clinical severity [109]. Exosomes carrying specific micro-RNAs (miRNAs) have been shown in the fibrotic late phase of ARDS. Compared with healthy volunteers, miR-425 was reduced in plasma exosomes from ARDS patients. To elucidate the role of this specific miRNA in the pathophysiology of the fibrotic phase of ARDS, an in vitro analysis showed that inhibition of miR-425 in a cell line of human fibroblasts induced collagen expression and promoted fibroblast proliferation [110].

An interesting aspect is the role of secretory autophagosomes (SAPs), double-membrane vesicles which can be regarded as EVs and exacerbate lung injury. SAPs were secreted by LPS-treated AMs and their intratracheal administration induced lung injury through IL-1β secretion [111]. EVs extracted from BALF of mice treated with intratracheal LPS showed an increased expression of miRNA-466. In vitro transfection of bone-marrow-derived macrophages with miRNA-466 increased the release of IL-1β after LPS stimulation. This finding suggests that miRNA-466 might have an important role in inflammasome activation, an essential mechanism of lung injury [112].

Specific miRNAs have also been characterized in EVs after lung viral infections. Compared with healthy volunteers, nine specific miRNAs have been found significantly upregulated or downregulated in the BALF of patients with Influenza H1N1-associated ARDS. In particular, miR-17-5p downregulated epithelial antiviral factors, such as Mx1 and E2F1 in lung epithelial cells infected by Influenza A virus, thus promoting a potential pro-viral effect [113]. A complex network of cell-to-cell interaction mediated by miRNAs in plasmatic EVs has been found in patients with COVID-19 associated ARDS. Downregulation of five specific EV-associated miRNAs in blood led to the activation of target molecules in ARDS patients, including IL-8 (CXCL8), which is a well-known mediator of neutrophil recruitment into the lung [114].

Pulmonary endothelium is a dynamic receptor-effector tissue sensor and responds to signals from extracellular environments. EVs are clue effectors of the interaction of lung endothelium with adjacent and circulating cells and mediators to modulate local immune-thrombosis, inflammatory cell adhesion, and integrity of alveolar units. In a rat model of LPS-induced lung injury, EVs of endothelial origin were higher in plasma [115] and their intravenous injection was characterized by higher levels of pro-inflammatory cytokines (IL-1β and TNFα), neutrophilic infiltration, and enhanced myeloperoxidase activity in the lungs [116]. Endothelial cells incubated with EVs had lower nitric oxide (NO) production, suggesting that EV-mediated impaired vasodilation (i.e., endothelial-derived microparticles) might have a role in ARDS pathogenesis [117].

Ex vivo ventilation and perfusion of human donor lungs rejected for clinical transplantation represents a platform to better understand lung pathophysiology and to test new treatments [118]. In an ex vivo human model, Liu et al. showed that EVs collected from the BALF of lungs challenged with E. coli bacteria were able to induce acute lung injury on naïve ventilated and perfused lungs, given through both intravenous and intrabronchial route. Most EVs were derived from endothelial cells and platelets, with a reduced contribution from monocytes, epithelial cells, and lymphocytes. Of interest, administration of hyaluronic acid reduced lung injury minimizing inflammation; the mechanism of this protection seems to be associated with a direct binding of hyaluronic acid to EVs with less uptake of EVs by human monocytes [119].

### 3.2. Role of Stem Cell-Derived Extracellular Vescicles as a Potential Therapeutic Tool in Sepsis-Associated ARDS

EVs derived from human MSCs were added in vitro to murine AMs that were subsequently administered intranasally to LPS-treated mice. Pre-treated AMs were protective against endotoxin-induced lung injury in terms of lower cell count, absolute neutrophils count, total proteins, and TNF-α in BALF [120].

The endosomal protein p18, which is expressed in the pulmonary endothelial cells, has a role in pulmonary endothelial integrity. Endothelial-derived EVs released from cells overexpressing the protein p18 protected the cell monolayer from LPS-induced permeability. The expression profile of miRNAs in EVs from p18 overexpressing cells was different compared with controls. In fact, specific miRNAs (i.e., miR-30a-5p, miR-96-5p, and miR-137-5p) attenuated or even completely blocked (i.e., let-7i-5p) endothelial permeability after LPS challenge in vitro [121]. These findings suggest that given the pivotal role of endothelium as a first barrier involved after tissue injury, endothelium-derived EVs may work as biomarkers of ongoing damage. Moreover, EVs derived from engineered endothelial cells may carry protective factors to stabilize the endothelial/epithelial barrier. A synthesis of pre-clinical in vitro and in vivo studies assessing the efficacy of MSC-derived EVs in ALI/ARDS models is shown in Table 2.

Recent data show that a pattern of circulating EVs characterize a protective endotype of ARDS. In a prospective observational study of 33 ARDS patients, long-term survivors had a circulating sub-phenotype characterized by MSC-derived EVs in blood containing higher RUNX1 isoform p66 (a transcription factor known to be involved in angiogenesis and MSCs proliferation), compared with patients who did not survive. The protective effect of RUNX1 isoform p66 seems to be associated with the induced ability of ECs to proliferate [139]. Higher levels of leukocyte-derived EVs in the blood of 52 ARDS patients were independently associated with improved adjusted survival at 28 days [140]. In addition, elevated EV concentration in plasma was independently associated with reduced risk of developing ARDS [141]. These data were expanded in a study showing that patients who underwent esophagectomy and further developed ARDS had higher concentration of endothelial-derived CD31 + EVs in BALF compared with healthy subjects. Considered together, these studies suggest that the secretome of monocytes and ECs play a role in ARDS pathogenesis and in identifying those patients who are at risk of developing the syndrome [108]. Table 3 showed a synthesis of clinical trials assessing the safety and efficacy of intravenous treatment with MSCs and MSC-derived EVs in human subjects with ARDS.

## 4. Potential Role of Extracellular Vesicles in Kidney-Lung Crosstalk and Future Therapeutic Perspectives

Inter-organ crosstalk between kidney and lung in critically ill patients has been the focus of intense research over the past years. On the one hand, AKI can affect the lung by altering the fluid and acid-base balance and through release and/or decreased clearance of inflammatory mediators. On the other hand, ARDS in a septic patient can worsen renal function through altered hemodynamics (hypoxia and hypercapnic acidosis, systemic congestion), neurohormonal dysregulation (activation of renin-angiotensin system, ADH, sympathetic system), biotrauma associated with mechanical ventilation and increased alveolar-capillary permeability (systemic release of IL-6 and other pro-inflammatory molecules), oxidative stress, accelerated tissue senescence. In particular, mechanical ventilation can add a further damage and around 30–60% of patients treated with it eventually require RRT [150].

**Figure 2:**
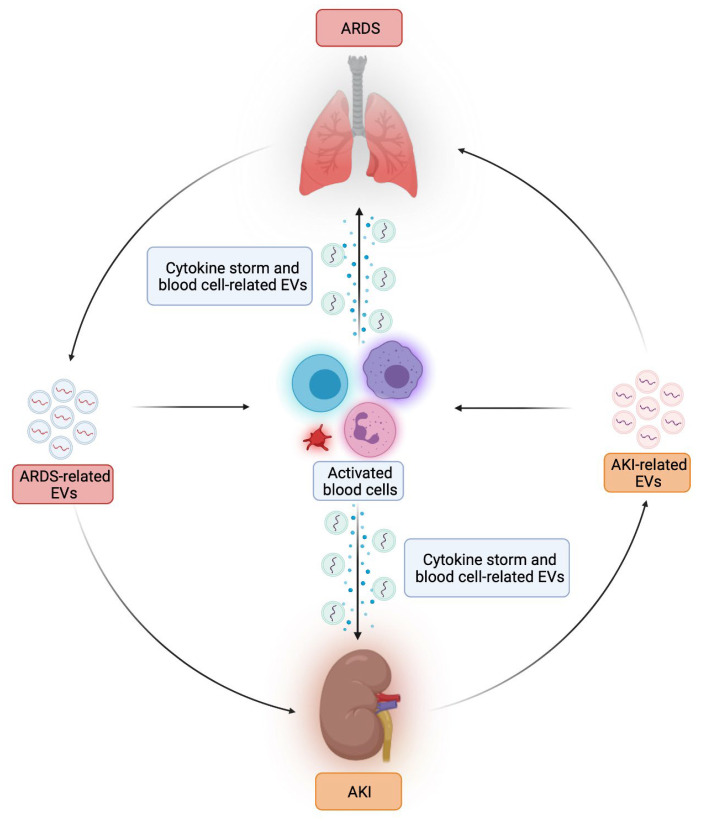
Potential role of EVs in kidney-lung crosstalk (Created with BioRender.com, accessed on 28 August 2022).

While significant progress has been made in elucidating mechanisms of this complex syndrome, the role of EVs in this setting is still undefined. However, it is biologically plausible that EVs are involved in the mutual exchange of deleterious mediators between kidney and lung, through endocrine actions (Figure 2). On the other hand, some evidence suggest that they may also exert protective actions and have the potential to limit organ damage [151].

**Figure 3:**
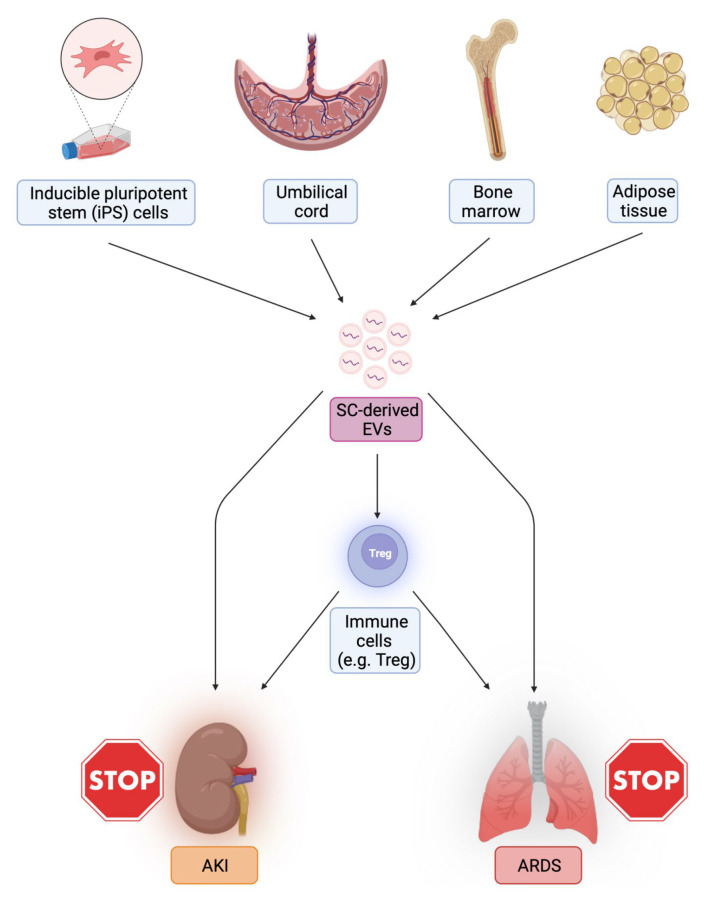
Potential therapeutic use of EVs to modulate kidney-lung crosstalk in sepsis and systemic inflammation (Created with BioRender.com, accessed on 28 August 2022).

On this basis, it is possible to envisage a potential therapeutic use of EVs to modulate inter-organ crosstalk in sepsis and dampen systemic inflammation. For example, an interesting option is the possibility of promoting AM polarization toward a pro-resolving M2 phenotype, with anti-inflammatory features. MSC-derived EVs can transfer mitochondria to AM and induce a switch from M1 to M2 phenotype [120] and specific EV-shuttled miRNAs (miR146a) have been associated with this process [152]. The presence of similar reparative mechanisms based on M2 macrophage both in the lung and in the kidney could pave the way to EV-based therapies that are effective on both organs [66] (Figure 3).

The pleiotropic actions of EVs make them a unique and versatile therapeutic tool, capable of simultaneous modulation of diverse pathogenetic aspects, ranging from endothelial dysfunction and thrombo-inflammation to tubular oxidative stress and macrophage phenotype. Increasing knowledge of EV actions on important activators of innate immunity, such as RTEC and AM, is strengthening the rationale for this therapy. EV-mediated crosstalk between RTEC and neighboring macrophages and between lung epithelial cells and AM is a key mechanism of damage in AKI and ALI, respectively. The potential of blunting this process with MSC-EVs or of employing EPC-EVs or endothelial-derived EVs to protect EC from LPS-induced permeability is intriguing as it represents a truly “etiopathogenetic” treatment.

Cyto-protection, tissue regeneration, and immunomodulation are three intertwined effects which appear to be partly shared in renal and pulmonary setting. Therefore, EV therapy may be a general tool to prevent and/or treat organ damage in sepsis, possibly even beyond kidney and lung.

Immunomodulatory properties of EVs may not only interfere with local mechanisms of damage, but also at a broader level with distant organ crosstalk and inflammatory dysregulation, a hallmark of sepsis. Expansion of T reg and inhibition of NK activity, for example, may shift the balance toward the control of inflammation.

Future clinical studies should cope with crucial aspects which could help expand the implementation of this therapy: the possibility of EVs manipulation to enrich them with drugs or protective miRNAs to target specific cell types (for example, ECs) of lung or kidney [153]; standardized methods for EV isolation and storage; preconditioning procedures to enhance their therapeutic properties; definition of dose-response relationship; availability of new biomarkers to assess effectiveness of MSC-derived EVs after administration [32,154].

## 5. Conclusions

EVs mediate a complex intercellular communication network in sepsis, shuttling a variety of key mediators, such as miRNAs. Paracrine or endocrine transfer of these molecules to target cells allows for the modulation of sets of genes, resulting in broad pro-inflammatory, pro-oxidant, and pro-apoptotic effects underlying microvascular dysfunction and multiple organ failure. This detrimental role is well documented both in septic AKI and ARDS and specific miRNAs are characterized which account for similar mechanisms of kidney and lung damage. On the other hand, organ-protective effects of SC-derived EVs have been reported in sepsis. These recapitulate cytoprotective, reparative and immunomodulatory properties of parental cells and have shown beneficial effects in experimental models of kidney and lung damage. This review summarizes the current knowledge on this dual, multi-faceted role and first emphasizes EV involvement in kidney-lung crosstalk. On this basis, EVs may represent a multi-level, comprehensive therapy of sepsis, with a potential for modulating pathogenetic mechanisms of both AKI and ALI in this setting.

## Figures and Tables

**Table 1:** Main pre-clinical studies assessing the efficacy of treatment with EVs in s-AKI models.

EV Strain and Model	Mechanisms	Treatment Effects	Reference
**EPC-EVs injected in a CLP rat model**	EV-carried miR-21-5p modulates RUNX1 axis	reduction in endothelial cell apoptosis and oxidative stress improved renal function and pathological lesions	[85]
**EPC-EVs injected in LPS-induced mouse model of s-AKI with MOD**	EV-carried mi-RNA-93-5p conferred endothelial protection via the KDM6B/H3K27me3/TNF-α axis	reduction in inflammation	[86]
**EPC-EVs injected in LPS-induced HK-2 cell injury**	EV-carried mi-RNA-93-5p alleviates LPS-induced HK-2 cell injury targeting miR-93-5p/OXSR1 axis	reduction in apoptosis, inflammation, and oxidative stress	[87]
**Rat model of s-AKI in vivo; LPS-induced sepsis model in HK-2 cells in vitro**	miR-22-3p downregulates HMGB1, p-p65, TLR4, and pro-inflammatory cytokines (IL-1β, IL-6, TNF-α), both in vivo and in vitro. It can also repress PTEN, a protein involved in mitophagy regulation	reduction in inflammation and apoptosis protection in mitochondrial function	[88]
**s-AKI mouse model through CLP**	Human MSC-EVs increased expression of miR-146b in kidney tissue and consequently reduced IRAK1 level and NF-κB activity	reduced inflammation improved morphological damage improved renal function and 72-h survival	[89]
**s-AKI mouse model through CLP**	AT-EVs activate SIRT1 signaling pathway blunting inflammation	improved renal function and survival	[90]
**s-AKI mouse model with remote ischemic pre-conditioning pre-treatment**	Exosomal miR-21 integrates into RTECs and targets PDCD4/NF-κB and PTEN/AKT pathways	reduced tubular apoptosis reduced inflammation	[91]
**s-AKI mouse model**	MSC-EVs from healthy controls transferred TFAM in recipient cells and restored TFAM-mtDNA complex stability, reversing mitochondrial oxidative phosphorylation defects after s-AKI	stabilization and reduced leakage of mtDNA reduced mitochondrial oxidative stress in injured RTECs reduced inflammation	[92]

**List of Abbreviations:** AT-EVs: Adipose tissue-derived extracellular vesicles; CLP: Cecal ligation and puncture; EPC: Endothelial progenitor cell; EV: Extracellular vesicle; HMGB-1: High mobility group box 1; IRAK1: Interleukin (IL)-1 receptor-associated kinase; LPS: Lipopolysaccharide; MOD: Multiple organ dysfunction; mtDNA: Mitochondrial DNA; MSCs: Mesenchymal stromal cell; OXSR1: Oxidative stress responsive kinase 1; PTEN: Phosphatase and tensin homologue; RTEC: Renal tubule epithelial cell; RUNX1: Runt-related transcription factor 1; s-AKI: Sepsis-associated acute kidney injury; TFAM: Mitochondrial transcription factor A; SIRT1: Sirtuin1.

**Table 2:** Synthesis of pre-clinical in vitro and in vivo studies assessing the efficacy of MSC-derived EVs in ALI/ARDS models.

Population	EV Strain	Lung Injury Model	Intervention Arms	Intervention Details	Mechanism Analyzed	Treatment Effects	Reference
**Rats**	WJMSC-EV	BLM IT	MSC-EV vs. Neg shRNA MSC-EV vs. HGF shRNA MSC-EV	MSC-EV IT	Apoptosis modulation via PI3K/AKT/mTOR signaling pathway	WBCs and neutrophils reduction in BALF TNF-α and IL-6 reduction in BALF EV therapeutic effects may be partly mediated by HGF mRNA	[122]
**Mice**	bm-MSCs naïve	LPS IP	bm-MSCs EV naïve	bm-MSCs exosomes IT 50 μg or bm-MSCs exosomes IT 100 μg	Glycolysis through HIF-1α inhibition Macrophage polarization	Improved histologic lung injury score and wet-to-dry ratio Increased oxygenation and reduced pro-inflammatory CKS in lung Improved survival Inhibition of glycolysis in lung and macrophage polarization	[123]
**Rats**	h-MSCs	*E. coli* IT	24 h CdM h-MSCs 48 h CdM h-MSCs vs. h-MSCs	CdM IV 300 μL	Macrophage phagocytosis	Enhanced survival Secretome did not reduce lung injury, BALF protein and neutrophils, pro-inflammatory CKs, *E. coli* counts	[124]
**Mice**	h-bm-MSCs	LPS IT	EV naïve vs. EV with dysfunctional mitochondria	EV from 5 × 10^5^ and 1 × 10^6^ MSCs	Restored mitochondrial function	Total protein and cell counts reduction in BALF Neutrophils reduction in BALF Therapeutic effects mediated by mitochondria	[125]
**Rats**	mu-MSCs	VILI	mu-MSCs vs. CdM	MSCs IV 10^6^, CdM IV 500 μL	IL-6 modulation	Enhanced arterial oxygenation Reduced neutrophil in BALF No effects on inflammatory cytokines CdM was not as effective as MSCs	[126]
**Mice**	hu-ADSCs	LPS IT	ADSCs from young donor (25 YO) vs. ADSCs from older donor (72 YO)	MSCs EV 100 μg IV 30 min after LPS	Macrophage polarization	Young MSCs-EV improved lung histology and reduced neutrophils in BALF Young MSCs-EV reduced IL1-β and increased IL-10 in BALF Young MSCs-EV favored the M2 macrophages’ phenotype	[127]
**Mice**	mu-MSCs	LPS IT	mu-MSCs CdM vs. mu-MSCs	CdM IT 30 µL	Macrophage polarization	BALF inflammation reduction Promotes the M2 anti-inflammatory AMs phenotype IGF-I mediated mechanism Attenuated lung inflammation	[128]
**Mice**	hu-bm-MSCs	LPS IT	Exo MSCs naïve vs. Exo MSCs NTF	Exo MSCs NTF IT 50 µL, 3 h after LPS 3 days until 72 h post-injury	Immune modulation balancing factors	Reduced histological damage and neutrophil accumulation Decreased IFN-γ, IL-6, TNF-α, and RANTES levels in BALF Improved oxygenation levels	[129]
**Mice**	bm-MSCs	SM SC	bm-MSCs-EV naïve	bm-MSCs-EV IV 20 mg/kg 24 h after injection of SM	Tight junction dysfunction and apoptosis inhibition	Reduced apoptosis in lung epithelial cells Repaired adherents and tight junction integrity Maintained vascular barrier integrity	[130]
**Mice**	hu-bm-MSCs	*E. coli* IT	EV vs. hu-bm-MSCs	EV IV 90 μL 4 h after injury	Enhanced macrophage-mediated bacterial phagocytosis	72-h survival increase through KGF mediation and reduced bacterial load Reduced neutrophils in BALF Decrease in bacterial load Anti-inflammatory IL-10 increased, pro-inflammatory TNFα reduced expression	[131]
**Mice**	mu-bm-MSCs	LPS IT LPS IP	mu-bm-MSCs EV vs. mu-bm-MSCs	EV IV released by 10^5^ cells	Effect of EV preconditioning with serum from ARDS mice EV effect in pulmonary or extrapulmonary ARDS	Reduced alveolar collapse and neutrophil cell counts in lung tissue Reduced higher total cell, macrophage, and neutrophil counts in BALF The effects of MSCs and EV differed according to ARDS aetiology Greater overall improvement from MSCs in comparison with EV	[132]
**Mice**	mu-MSCs	LPS IT	mu-MSCs CdM	CdM IV 200 µL	Neutrophil apoptosis	Decrease in neutrophil accumulation in lung tissues Pro-inflammatory CKs reduction in lung tissues Enhancement of apoptosis in BALF neutrophils	[133]
**Mice**	mu-bm-MSCs	LPS IT	HLMVECs vs. Neg SiRNA h-MSCs vs. Ang-1 SiRNA h-MSCs	HLMVECs IT 2 × 10^5^	Macrophage polarization	Ang-1 mRNA mediates the therapeutic effects of EV Decreased lung inflammation and pulmonary oedema Pro-inflammatory MIP2 reduction in BALF Reduced albumin levels in BALF	[134]
**Rats**	hu-MSCs naive	E. coli IT	IFNγ-primed EV vs. naïve EV	EV IT 100 × 10^6^/Kg	Macrophage phagocytosis	Improved survival Attenuated lung injury Restoration of NO synthase Enhancement of phagocytosis and bacterial killing	[135]
**Mice**	hu-MSCs	LPS IT	hu-MSCs EV naïve vs. hu-MSCs EV + autophagy inhibitor	hu-MSCs exosomes IT 50 μg 4 h after LPS	Autophagy	Protein reduction in BALF IL1-β, IL-6, IL-17, and MCP1 reduction in BALF Autophagy might mediate ALI recovery	[136]
**Rats**	bm-EPCs	LPS IT	EPC-EV vs. EPC-EV + GW4869	EPC-EV IV 10 μg	miR-126-mediated modulation of RAF/ERK signaling pathway	Improved arterial oxygenation and lung histology Decreased lung wet-to-dry ratio Decreased total protein in BALF Endothelial function improvement	[137]
**Mice**	hu-MSCs	E. coli IT	MSC-EV IT vs. MSC-EV IV vs. KGF siRNA- Pre-treated	MSC-EV 30 µL IT	KGF protein expression through mRNA modulation	BALF inflamattory response reduction at 48 h Decrease in extravascular lung water Restored protein permeability over 24 h BALF KGF protein increased	[138]

**List of Abbreviations**: ADSCs: Adipose-derived mesenchymal stem cells; ALI: Acute lung injury; Ang: Angiopoietin; ARDS: Acute respiratory distress syndrome; BLM: Bleomycin; bmEPCs: Bone marrow-derived endothelial progenitor cells; bm-MSCs: Bone marrow MSCs; EPC: Endothelial progenitor cells; EVs: Extra-cellular vesicles; Exo MSCs NTF: Small EVs derived from NurOwn MSC-NTF cells; Exo MSCs: EVs derived from undifferentiated MSCs; h-: Human; HIF-1a: Hypoxia-inducible factor 1-alpha; HLMVECs: Human lung microvascular endothelial cells; hu-: Human umbilical; IN: Intranasal; IP: Intraperitoneal; IT: Intratracheal; IV: Intravenous; LPS: Lipopolysaccharide; LXA4: Lipoxin A4; MCP-1: Monocyte chemoattractant protein 1; mu-: Murine; NTF: Neurotrophic and immunomodulatory factors secreting MSCs; PBMC: Peripheral blood mononuclear cell; PBS: Phosphate-buffered saline; RANTES: Regulated on activation normal T-cell expressed and secreted; SM: Sulphur mustard; VILI: Ventilator-induced lung injury; WJMSC: Wharton’s Jelly mesenchymal stem cells.

**Table 3:** Synthesis of clinical trials assessing the safety and efficacy of intravenous treatment with MSCs and MSC-derived EVs in human subjects with ARDS.

Study Design	*n*	Clinical Context and Inclusion Criteria	Intervention	Treatment Effect	Reference
Prospective interventional (ex vivo)	*n* = 37	EVLP in rejected lungs with *E. coli*-induced pneumonia MSCs pre-treatment with TLR-3 agonist (TLR3+)	10 µL = EV secreted by 10^6^ MSCs Four treatment arms: (1) 200 µL MSC EVs (2) 400 µL MSC EVs (3) 200 µL TLR-3+ MSC EVs (4) 200 µL NHLF EV	Increased alveolar fluid clearance Reduced lung protein permeability Enhanced antimicrobial activity Reduced PAP	[142]
Prospective interventional (single arm)	*n* = 24	COVID-19 patients Dyspnoea for >72 h Down trending P/F	15 mL of ExoFlo^TM^ (bm-MSC exosomes)	Oxygenation and lymphocyte count improvement Serum D-dimer, C-reactive protein, and ferritin reduction NC decrease/LC increase No EV-related SAE	[143]
RCT	*n* = 40 (*n* = 20 C *n* = 20 T)	Critically ill COVID-19 patients with pneumonia Patients with leuco- and lymphopenia	10^6^/kg UC-MSCs (T arm) vs. NS (C arm)	Improved survival rate in T arm (2.5 times overall, 4.5 times in patients with comorbidities) Significantly decreased IL-6 in the recovered patients in the T arm	[144]
RCT	*n* = 24 (*n* = 12 C *n* = 12 T)	SpO_2_ ≤ 94% at room air P/F < 300 mmHg Bilateral infiltrates on CXR or bilateral ground glass opacities on a chest CT scan	(100 ± 20) × 10^6^ UC-MSCs × 2 administrations (T arm) vs. vehicle solution NS (C arm)	No difference in infusion-associated AE between groups No SAE associated with MSCs Improved survival, SAE-free survival, and time-to-recovery in T arm GM-CSF, IFNγ, IL-5, IL-6, IL-7, TNFα, TNFβ, PDGF-BB, and RANTES (T arm, day 6)	[145]
RCT	*n* = 12 (*n* = 6 C *n* = 6 T)	ARDS patients P/F < 200 mmHg	10^6^ cells/kg adipose-derived MSCs	No infusion toxicities or SAE in the T arm Serum SP-D day 5 lower than day 0 in T arm	[146]
Prospective Phase 1 CT	*n* = 9	ARDS patients P/F < 200 mmHg	Three treatment arms: (1) 1 × 10^6^ UC-MSCs/kg (2) 5 × 10^6^ UC-MSCs/kg (3) 1 × 10^7^ UC-MSCs/kg	No SAE in the three arms Minor non-life-threatening AE in 3 patients	[147]
Open label clinical trial	*n* = 61 (*n* = 44 C *n* = 17 T)	H7N9 ARDS patients P/F < 200 mmHg	Three treatment arms, administration of 1 × 10^6^ BMD-MSCs/kg: (1) 3 infusions, early stage (2) 3 infusions, late stage (3) 4 infusions, late stage	Higher survival rate T group No SAE in a 5-year follow-up	[148]
Phase 1/2 multicentre RCT Cohort 1 and 2	*n* = 6 (*n* = 3 C1 *n* = 3 C2)	ARDS patients P/F < 200 mmHg	Two treatment arms: (1) 300 × 10^6^ MAPC (2) 900 × 10^6^ MAPC	No AE and SAE related to treatment	[149]
Phase 1/2 multicentre RCT Cohort 3	*n* = 30 (*n* = 10 C *n* = 20 T)	ARDS patients P/F < 200 mmHg	900 × 10^6^ MAPC (T arm) vs. placebo (C arm)	One possibly related, non-serious AE in T arm	

**List of Abbreviations**: MSCs: Mesenchymal stem cells; EVs: Extracellular vesicles; EVLP: Ex vivo lung perfusion; IV: Intravenous; TLR-3: Toll-like receptor 3; NHLF: Normal human lung fibroblasts; PAP: Pulmonary arterial pressure; P/F: PaO_2_/FiO_2_ ratio; bm-MSCs: Bone marrow MSCs; NC: Absolute neutrophil count; LC: Absolute lymphocyte count; SAE: Serious adverse events; RCT: Randomized controlled trial; C: Control arm; T: Treatment arm; UC-MSCs: Umbilical cord MSCs; NS: Normal saline; SPO2: Peripheral arterial oxygen saturation; CXR: Chest X-ray; CT: Computed tomography; AE: Adverse events; ARDS: Acute respiratory distress syndrome; SP-D: Surfactant protein D; BMD-MSCs: Blood menstrual-derived MSCs; MAPC: Multipotent adult progenitor cells.

## Data Availability

Not applicable.

## Abbreviations

AKI	acute kidney injury
ALI	acute lung injury
BALF	bronco-alveolar lavage fluid
CKD	chronic kidney disease
CLP	cecal ligation and puncture
DAMP	damage-associated molecular pattern
DC	dendritic cell
EC	endothelial cell
EMT	epithelial-to-mesenchymal transition
EndMT	endothelial-to-mesenchymal transition
EPC	endothelial progenitor cell
EV	extracellular vesicles
ICU	intensive care unit
IL	interleukin
IRI	ischemia-reperfusion injury
LPS	lipopolysaccharide
mDNA	mitochondrial DNA
MOD	multi-organ dysfunction
miRNA	microRNA
NEAT-1	nuclear-enriched abundant transcript 1
NRF-2	nuclear factor erythroid 2-related factor 2
PAMP	pathogen-associated molecular pattern
PMN	polymorphonuclear cell
PMT	pericyte-to-mesenchymal transition
ROS	reactive oxygen species
RRT	renal replacement therapy
RTEC	renal tubular epithelial cells
RUNX1	runt-related transcription factor 1 axis
s-AKI	sepsis-associated AKI
SASP	senescence-associated secretory phenotype
SC	stem cell
SOCS-1	suppressor of cytokine signaling-1
TGFβ-1	transforming growth factor β-1
TLR	Toll-like receptors
TXNIP	thioredoxin-interacting protein

## References

[1] Raposo G., Stoorvogel W. (2013). Extracellular Vesicles: Exosomes, Microvesicles, and Friends. J. Cell Biol..

[2] Cocucci E., Meldolesi J. (2015). Ectosomes and Exosomes: Shedding the Confusion between Extracellular Vesicles. Trends Cell Biol..

[3] Bobrie A., Colombo M., Krumeich S., Raposo G., Théry C. (2012). Diverse Subpopulations of Vesicles Secreted by Different Intracellular Mechanisms Are Present in Exosome Preparations Obtained by Differential Ultracentrifugation. J. Extracell. Vesicles.

[4] Abels E.R., Breakefield X.O. (2016). Introduction to Extracellular Vesicles: Biogenesis, RNA Cargo Selection, Content, Release, and Uptake. Cell. Mol. Neurobiol..

[5] Doyle L.M., Wang M.Z. (2019). Overview of Extracellular Vesicles, Their Origin, Composition, Purpose, and Methods for Exosome Isolation and Analysis. Cells.

[6] Hristov M., Erl W., Linder S., Weber P.C. (2004). Apoptotic Bodies from Endothelial Cells Enhance the Number and Initiate the Differentiation of Human Endothelial Progenitor Cells in Vitro. Blood.

[7] Van Niel G., D’Angelo G., Raposo G. (2018). Shedding Light on the Cell Biology of Extracellular Vesicles. Nat. Rev. Mol. Cell Biol..

[8] Urabe F., Kosaka N., Ito K., Kimura T., Egawa S., Ochiya T. (2020). Extracellular Vesicles as Biomarkers and Therapeutic Targets for Cancer. Am. J. Physiol. Cell Physiol..

[9] Ratajczak J., Miekus K., Kucia M., Zhang J., Reca R., Dvorak P., Ratajczak M.Z. (2006). Embryonic Stem Cell-Derived Microvesicles Reprogram Hematopoietic Progenitors: Evidence for Horizontal Transfer of MRNA and Protein Delivery. Leukemia.

[10] Valadi H., Ekström K., Bossios A., Sjöstrand M., Lee J.J., Lötvall J.O. (2007). Exosome-Mediated Transfer of MRNAs and MicroRNAs Is a Novel Mechanism of Genetic Exchange between Cells. Nat. Cell Biol..

[11] Mathieu M., Martin-Jaular L., Lavieu G., Théry C. (2019). Specificities of Secretion and Uptake of Exosomes and Other Extracellular Vesicles for Cell-to-Cell Communication. Nat. Cell Biol..

[12] Kodam S.P., Ullah M. (2021). Diagnostic and Therapeutic Potential of Extracellular Vesicles. Technol. Cancer Res. Treat..

[13] Claridge B., Lozano J., Poh Q.H., Greening D.W. (2021). Development of Extracellular Vesicle Therapeutics: Challenges, Considerations, and Opportunities. Front. Cell Dev. Biol..

[14] Rady D., Abbass M.M.S., El-Rashidy A.A., El Moshy S., Radwan I.A., Dörfer C.E., Fawzy El-Sayed K.M. (2020). Mesenchymal Stem/Progenitor Cells: The Prospect of Human Clinical Translation. Stem Cells Int..

[15] Salybekov A.A., Kunikeyev A.D., Kobayashi S., Asahara T. (2021). Latest Advances in Endothelial Progenitor Cell-Derived Extracellular Vesicles Translation to the Clinic. Front. Cardiovasc. Med..

[16] Terriaca S., Fiorelli E., Scioli M.G., Fabbri G., Storti G., Cervelli V., Orlandi A. (2021). Endothelial Progenitor Cell-Derived Extracellular Vesicles: Potential Therapeutic Application in Tissue Repair and Regeneration. Int. J. Mol. Sci..

[17] Cantaluppi V., Medica D., Mannari C., Stiaccini G., Figliolini F., Dellepiane S., Quercia A.D., Migliori M., Panichi V., Giovannini L. (2015). Endothelial Progenitor Cell-Derived Extracellular Vesicles Protect from Complement-Mediated Mesangial Injury in Experimental Anti-Thy1.1 Glomerulonephritis. Nephrol. Dial. Transplant..

[18] Qiu P., Zhou J., Zhang J., Dong Y., Liu Y. (2021). Exosome: The Regulator of the Immune System in Sepsis. Front. Pharmacol..

[19] Singer M., Deutschman C.S., Seymour C.W., Shankar-Hari M., Annane D., Bauer M., Bellomo R., Bernard G.R., Chiche J.-D., Coopersmith C.M. (2016). The Third International Consensus Definitions for Sepsis and Septic Shock (Sepsis-3). JAMA.

[20] Rudd K.E., Johnson S.C., Agesa K.M., Shackelford K.A., Tsoi D., Kievlan D.R., Colombara D.V., Ikuta K.S., Kissoon N., Finfer S. (2020). Global, Regional, and National Sepsis Incidence and Mortality, 1990–2017: Analysis for the Global Burden of Disease Study. Lancet.

[21] Kaukonen K.-M., Bailey M., Suzuki S., Pilcher D., Bellomo R. (2014). Mortality Related to Severe Sepsis and Septic Shock among Critically Ill Patients in Australia and New Zealand, 2000–2012. JAMA.

[22] Vincent J.-L., Marshall J.C., Namendys-Silva S.A., François B., Martin-Loeches I., Lipman J., Reinhart K., Antonelli M., Pickkers P., Njimi H. (2014). Assessment of the Worldwide Burden of Critical Illness: The Intensive Care over Nations (ICON) Audit. Lancet Respir. Med..

[23] Rossaint J., Zarbock A. (2015). Pathogenesis of Multiple Organ Failure in Sepsis. Crit. Rev. Immunol..

[24] May C.N., Bellomo R., Lankadeva Y.R. (2021). Therapeutic Potential of Megadose Vitamin C to Reverse Organ Dysfunction in Sepsis and COVID-19. Br. J. Pharmacol..

[25] Annane D., Bellissant E., Sebille V., Lesieur O., Mathieu B., Raphael J.C., Gajdos P. (1998). Impaired Pressor Sensitivity to Noradrenaline in Septic Shock Patients with and without Impaired Adrenal Function Reserve. Br. J. Clin. Pharmacol..

[26] Kellum J.A., Lameire N., KDIGO AKI Guideline Work Group (2013). Diagnosis, Evaluation, and Management of Acute Kidney Injury: A KDIGO Summary (Part 1). Crit. Care.

[27] Poston J.T., Koyner J.L. (2019). Sepsis Associated Acute Kidney Injury. BMJ.

[28] Kellum J.A., Chawla L.S., Keener C., Singbartl K., Palevsky P.M., Pike F.L., Yealy D.M., Huang D.T., Angus D.C., ProCESS and ProGReSS-AKI Investigators (2016). The Effects of Alternative Resuscitation Strategies on Acute Kidney Injury in Patients with Septic Shock. Am. J. Respir. Crit. Care Med..

[29] Mehta R.L., Bouchard J., Soroko S.B., Ikizler T.A., Paganini E.P., Chertow G.M., Himmelfarb J., Program to Improve Care in Acute Renal Disease (PICARD) Study Group (2011). Sepsis as a Cause and Consequence of Acute Kidney Injury: Program to Improve Care in Acute Renal Disease. Intensive Care Med..

[30] Lee S.A., Cozzi M., Bush E.L., Rabb H. (2018). Distant Organ Dysfunction in Acute Kidney Injury: A Review. Am. J. Kidney Dis..

[31] Kurzhagen J.T., Dellepiane S., Cantaluppi V., Rabb H. (2020). AKI: An Increasingly Recognized Risk Factor for CKD Development and Progression. J. Nephrol..

[32] Quaglia M., Merlotti G., Colombatto A., Bruno S., Stasi A., Franzin R., Castellano G., Grossini E., Fanelli V., Cantaluppi V. (2022). Stem Cell-Derived Extracellular Vesicles as Potential Therapeutic Approach for Acute Kidney Injury. Front. Immunol..

[33] Wu X., Liu Y., Wei W., Liu M.-L. (2019). Extracellular Vesicles in Autoimmune Vasculitis—Little Dirts Light the Fire in Blood Vessels. Autoimmun. Rev..

[34] Mariano F., Cantaluppi V., Stella M., Romanazzi G.M., Assenzio B., Cairo M., Biancone L., Triolo G., Ranieri V.M., Camussi G. (2008). Circulating Plasma Factors Induce Tubular and Glomerular Alterations in Septic Burns Patients. Crit. Care.

[35] Gómez H., Kellum J.A. (2016). Sepsis-Induced Acute Kidney Injury. Curr. Opin. Crit. Care.

[36] Cantaluppi V., Biancone L., Quercia A., Deregibus M.C., Segoloni G., Camussi G. (2013). Rationale of Mesenchymal Stem Cell Therapy in Kidney Injury. Am. J. Kidney Dis..

[37] Bussolati B., Camussi G. (2015). Therapeutic Use of Human Renal Progenitor Cells for Kidney Regeneration. Nat. Rev. Nephrol..

[38] Franzin R., Stasi A., Ranieri E., Netti G.S., Cantaluppi V., Gesualdo L., Stallone G., Castellano G. (2021). Targeting Premature Renal Aging: From Molecular Mechanisms of Cellular Senescence to Senolytic Trials. Front. Pharmacol..

[39] Franzin R., Stasi A., Fiorentino M., Stallone G., Cantaluppi V., Gesualdo L., Castellano G. (2020). Inflammaging and Complement System: A Link Between Acute Kidney Injury and Chronic Graft Damage. Front. Immunol..

[40] Peerapornratana S., Manrique-Caballero C.L., Gómez H., Kellum J.A. (2019). Acute Kidney Injury from Sepsis: Current Concepts, Epidemiology, Pathophysiology, Prevention and Treatment. Kidney Int..

[41] Souza A.C.P., Yuen P.S.T., Star R.A. (2015). Microparticles: Markers and Mediators of Sepsis-Induced Microvascular Dysfunction, Immunosuppression, and AKI. Kidney Int..

[42] Karpman D., Tontanahal A. (2021). Extracellular Vesicles in Renal Inflammatory and Infectious Diseases. Free Radic. Biol. Med..

[43] Zheng D., Zhang J., Zhang Z., Kuang L., Zhu Y., Wu Y., Xue M., Zhao H., Duan C., Liu L. (2020). Endothelial Microvesicles Induce Pulmonary Vascular Leakage and Lung Injury During Sepsis. Front. Cell Dev. Biol..

[44] Mastronardi M.L., Mostefai H.A., Meziani F., Martínez M.C., Asfar P., Andriantsitohaina R. (2011). Circulating Microparticles from Septic Shock Patients Exert Differential Tissue Expression of Enzymes Related to Inflammation and Oxidative Stress. Crit. Care Med..

[45] Mortaza S., Martinez M.C., Baron-Menguy C., Burban M., de la Bourdonnaye M., Fizanne L., Pierrot M., Calès P., Henrion D., Andriantsitohaina R. (2009). Detrimental Hemodynamic and Inflammatory Effects of Microparticles Originating from Septic Rats. Crit. Care Med..

[46] Raeven P., Zipperle J., Drechsler S. (2018). Extracellular Vesicles as Markers and Mediators in Sepsis. Theranostics.

[47] Iba T., Ogura H. (2018). Role of Extracellular Vesicles in the Development of Sepsis-Induced Coagulopathy. J. Intensive Care.

[48] Tőkés-Füzesi M., Woth G., Ernyey B., Vermes I., Mühl D., Bogár L., Kovács G.L. (2013). Microparticles and Acute Renal Dysfunction in Septic Patients. J. Crit. Care.

[49] Ma S., Evans R.G., Iguchi N., Tare M., Parkington H.C., Bellomo R., May C.N., Lankadeva Y.R. (2019). Sepsis-Induced Acute Kidney Injury: A Disease of the Microcirculation. Microcirculation.

[50] Nadim M.K., Forni L.G., Mehta R.L., Connor M.J., Liu K.D., Ostermann M., Rimmelé T., Zarbock A., Bell S., Bihorac A. (2020). COVID-19-Associated Acute Kidney Injury: Consensus Report of the 25th Acute Disease Quality Initiative (ADQI) Workgroup. Nat. Rev. Nephrol..

[51] Legrand M., Bell S., Forni L., Joannidis M., Koyner J.L., Liu K., Cantaluppi V. (2021). Pathophysiology of COVID-19-Associated Acute Kidney Injury. Nat. Rev. Nephrol..

[52] Petruk G., Puthia M., Petrlova J., Samsudin F., Strömdahl A.-C., Cerps S., Uller L., Kjellström S., Bond P.J., Schmidtchen A.A. (2020). SARS-CoV-2 Spike Protein Binds to Bacterial Lipopolysaccharide and Boosts Proinflammatory Activity. J. Mol. Cell Biol..

[53] Cappellano G., Raineri D., Rolla R., Giordano M., Puricelli C., Vilardo B., Manfredi M., Cantaluppi V., Sainaghi P.P., Castello L. (2021). Circulating Platelet-Derived Extracellular Vesicles Are a Hallmark of SARS-CoV-2 Infection. Cells.

[54] Barberis E., Vanella V.V., Falasca M., Caneapero V., Cappellano G., Raineri D., Ghirimoldi M., de Giorgis V., Puricelli C., Vaschetto R. (2021). Circulating Exosomes Are Strongly Involved in SARS-CoV-2 Infection. Front. Mol. Biosci..

[55] Janiszewski M., Do Carmo A.O., Pedro M.A., Silva E., Knobel E., Laurindo F.R.M. (2004). Platelet-Derived Exosomes of Septic Individuals Possess Proapoptotic NAD(P)H Oxidase Activity: A Novel Vascular Redox Pathway. Crit. Care Med..

[56] Burger D., Turner M., Munkonda M.N., Touyz R.M. (2016). Endothelial Microparticle-Derived Reactive Oxygen Species: Role in Endothelial Signaling and Vascular Function. Oxidative Med. Cell. Longev..

[57] Dolmatova E.V., Wang K., Mandavilli R., Griendling K.K. (2021). The Effects of Sepsis on Endothelium and Clinical Implications. Cardiovasc. Res..

[58] Burger D., Montezano A.C., Nishigaki N., He Y., Carter A., Touyz R.M. (2011). Endothelial Microparticle Formation by Angiotensin II Is Mediated via Ang II Receptor Type I/NADPH Oxidase/Rho Kinase Pathways Targeted to Lipid Rafts. Arterioscler. Thromb. Vasc. Biol..

[59] Sonoda H., Lee B.R., Park K.-H., Nihalani D., Yoon J.-H., Ikeda M., Kwon S.-H. (2019). MiRNA Profiling of Urinary Exosomes to Assess the Progression of Acute Kidney Injury. Sci. Rep..

[60] Toro J., Manrique-Caballero C.L., Gómez H. (2021). Metabolic Reprogramming and Host Tolerance: A Novel Concept to Understand Sepsis-Associated AKI. J. Clin. Med..

[61] Cantaluppi V., Quercia A.D., Dellepiane S., Ferrario S., Camussi G., Biancone L. (2014). Interaction between Systemic Inflammation and Renal Tubular Epithelial Cells. Nephrol. Dial. Transplant..

[62] Emma F., Montini G., Parikh S.M., Salviati L. (2016). Mitochondrial Dysfunction in Inherited Renal Disease and Acute Kidney Injury. Nat. Rev. Nephrol..

[63] Ralto K.M., Rhee E.P., Parikh S.M. (2020). NAD+ Homeostasis in Renal Health and Disease. Nat. Rev. Nephrol..

[64] Youn Y.-J., Shrestha S., Lee Y.-B., Kim J.-K., Lee J.H., Hur K., Mali N.M., Nam S.-W., Kim S.-H., Lee S. (2021). Neutrophil-Derived Trail Is a Proinflammatory Subtype of Neutrophil-Derived Extracellular Vesicles. Theranostics.

[65] Essandoh K., Li Y., Huo J., Fan G.-C. (2016). MiRNA-Mediated Macrophage Polarization and Its Potential Role in the Regulation of Inflammatory Response. Shock.

[66] Hu Q., Lyon C.J., Fletcher J.K., Tang W., Wan M., Hu T.Y. (2021). Extracellular Vesicle Activities Regulating Macrophage- and Tissue-Mediated Injury and Repair Responses. Acta Pharm. Sin. B.

[67] Lv L.-L., Feng Y., Wu M., Wang B., Li Z.-L., Zhong X., Wu W.-J., Chen J., Ni H.-F., Tang T.-T. (2020). Exosomal MiRNA-19b-3p of Tubular Epithelial Cells Promotes M1 Macrophage Activation in Kidney Injury. Cell Death Differ..

[68] Wang Z.-W., Zhu X. (2019). Exosomal MiR-19b-3p Communicates Tubular Epithelial Cells and M1 Macrophage. Cell Death Dis..

[69] Li Z.-L., Lv L.-L., Tang T.-T., Wang B., Feng Y., Zhou L.-T., Cao J.-Y., Tang R.-N., Wu M., Liu H. (2019). HIF-1α Inducing Exosomal MicroRNA-23a Expression Mediates the Cross-Talk between Tubular Epithelial Cells and Macrophages in Tubulointerstitial Inflammation. Kidney Int..

[70] Lv L.-L., Feng Y., Wen Y., Wu W.-J., Ni H.-F., Li Z.-L., Zhou L.-T., Wang B., Zhang J.-D., Crowley S.D. (2018). Exosomal CCL2 from Tubular Epithelial Cells Is Critical for Albumin-Induced Tubulointerstitial Inflammation. J. Am. Soc. Nephrol..

[71] Juan C.-X., Mao Y., Cao Q., Chen Y., Zhou L.-B., Li S., Chen H., Chen J.-H., Zhou G.-P., Jin R. (2021). Exosome-Mediated Pyroptosis of MiR-93-TXNIP-NLRP3 Leads to Functional Difference between M1 and M2 Macrophages in Sepsis-Induced Acute Kidney Injury. J. Cell Mol. Med..

[72] Ye Z., Zhang L., Li R., Dong W., Liu S., Li Z., Liang H., Wang L., Shi W., Malik A.B. (2019). Caspase-11 Mediates Pyroptosis of Tubular Epithelial Cells and Septic Acute Kidney Injury. Kidney Blood Press. Res..

[73] Gildea J.J., Seaton J.E., Victor K.G., Reyes C.M., Bigler Wang D., Pettigrew A.C., Courtner C.E., Shah N., Tran H.T., van Sciver R.E. (2014). Exosomal Transfer from Human Renal Proximal Tubule Cells to Distal Tubule and Collecting Duct Cells. Clin. Biochem..

[74] Cricrì G., Bellucci L., Montini G., Collino F. (2021). Urinary Extracellular Vesicles: Uncovering the Basis of the Pathological Processes in Kidney-Related Diseases. Int. J. Mol. Sci..

[75] Fatima F., Ekstrom K., Nazarenko I., Maugeri M., Valadi H., Hill A.F., Camussi G., Nawaz M. (2017). Non-Coding RNAs in Mesenchymal Stem Cell-Derived Extracellular Vesicles: Deciphering Regulatory Roles in Stem Cell Potency, Inflammatory Resolve, and Tissue Regeneration. Front. Genet..

[76] Tsuji K., Kitamura S., Wada J. (2018). Secretomes from Mesenchymal Stem Cells against Acute Kidney Injury: Possible Heterogeneity. Stem Cells Int..

[77] Li J.-K., Yang C., Su Y., Luo J.-C., Luo M.-H., Huang D.-L., Tu G.-W., Luo Z. (2021). Mesenchymal Stem Cell-Derived Extracellular Vesicles: A Potential Therapeutic Strategy for Acute Kidney Injury. Front. Immunol..

[78] Kaushal G.P., Shah S.V. (2016). Autophagy in Acute Kidney Injury. Kidney Int..

[79] Grange C., Skovronova R., Marabese F., Bussolati B. (2019). Stem Cell-Derived Extracellular Vesicles and Kidney Regeneration. Cells.

[80] Maccario R., Podestà M., Moretta A., Cometa A., Comoli P., Montagna D., Daudt L., Ibatici A., Piaggio G., Pozzi S. (2005). Interaction of Human Mesenchymal Stem Cells with Cells Involved in Alloantigen-Specific Immune Response Favors the Differentiation of CD4+ T-Cell Subsets Expressing a Regulatory/Suppressive Phenotype. Haematologica.

[81] Zou X., Gu D., Zhang G., Zhong L., Cheng Z., Liu G., Zhu Y. (2016). NK Cell Regulatory Property Is Involved in the Protective Role of MSC-Derived Extracellular Vesicles in Renal Ischemic Reperfusion Injury. Hum. Gene Ther..

[82] Gatti S., Bruno S., Deregibus M.C., Sordi A., Cantaluppi V., Tetta C., Camussi G. (2011). Microvesicles Derived from Human Adult Mesenchymal Stem Cells Protect against Ischaemia-Reperfusion-Induced Acute and Chronic Kidney Injury. Nephrol. Dial. Transplant..

[83] Ferguson S.W., Wang J., Lee C.J., Liu M., Neelamegham S., Canty J.M., Nguyen J. (2018). The MicroRNA Regulatory Landscape of MSC-Derived Exosomes: A Systems View. Sci. Rep..

[84] Wang S.-Y., Hong Q., Zhang C.-Y., Yang Y.-J., Cai G.-Y., Chen X.-M. (2019). MiRNAs in Stem Cell-Derived Extracellular Vesicles for Acute Kidney Injury Treatment: Comprehensive Review of Preclinical Studies. Stem Cell Res. Ther..

[85] Zhang Y., Huang H., Liu W., Liu S., Wang X.Y., Diao Z.L., Zhang A.H., Guo W., Han X., Dong X. (2021). Endothelial Progenitor Cells-Derived Exosomal MicroRNA-21-5p Alleviates Sepsis-Induced Acute Kidney Injury by Inhibiting RUNX1 Expression. Cell Death Dis..

[86] He Z., Wang H., Yue L. (2020). Endothelial Progenitor Cells-Secreted Extracellular Vesicles Containing MicroRNA-93-5p Confer Protection against Sepsis-Induced Acute Kidney Injury via the KDM6B/H3K27me3/TNF-α Axis. Exp. Cell Res..

[87] Li H., Zhang X., Wang P., Zhou X., Liang H., Li C. (2021). Knockdown of Circ-FANCA Alleviates LPS-Induced HK2 Cell Injury via Targeting MiR-93-5p/OXSR1 Axis in Septic Acute Kidney Injury. Diabetol. Metab. Syndr..

[88] Wang X., Wang Y., Kong M., Yang J. (2020). MiR-22-3p Suppresses Sepsis-Induced Acute Kidney Injury by Targeting PTEN. Biosci. Rep..

[89] Zhang R., Zhu Y., Li Y., Liu W., Yin L., Yin S., Ji C., Hu Y., Wang Q., Zhou X. (2020). Human Umbilical Cord Mesenchymal Stem Cell Exosomes Alleviate Sepsis-Associated Acute Kidney Injury via Regulating MicroRNA-146b Expression. Biotechnol. Lett..

[90] Gao F., Zuo B., Wang Y., Li S., Yang J., Sun D. (2020). Protective Function of Exosomes from Adipose Tissue-Derived Mesenchymal Stem Cells in Acute Kidney Injury through SIRT1 Pathway. Life Sci..

[91] Pan T., Jia P., Chen N., Fang Y., Liang Y., Guo M., Ding X. (2019). Delayed Remote Ischemic Preconditioning ConfersRenoprotection against Septic Acute Kidney Injury via Exosomal MiR-21. Theranostics.

[92] Zhao M., Liu S., Wang C., Wang Y., Wan M., Liu F., Gong M., Yuan Y., Chen Y., Cheng J. (2021). Mesenchymal Stem Cell-Derived Extracellular Vesicles Attenuate Mitochondrial Damage and Inflammation by Stabilizing Mitochondrial DNA. ACS Nano.

[93] Chong C.-R., Chan W.P.A., Nguyen T.H., Liu S., Procter N.E.K., Ngo D.T., Sverdlov A.L., Chirkov Y.Y., Horowitz J.D. (2014). Thioredoxin-Interacting Protein: Pathophysiology and Emerging Pharmacotherapeutics in Cardiovascular Disease and Diabetes. Cardiovasc. Drugs Ther..

[94] Yang J., Wu L., Liu S., Hu X., Wang Q., Fang L. (2021). Long Non-Coding RNA NEAT1 Promotes Lipopolysaccharide-Induced Injury in Human Tubule Epithelial Cells by Regulating MiR-93-5p/TXNIP Axis. Med. Microbiol. Immunol..

[95] Ranieri V.M., Rubenfeld G.D., Thompson B.T., Ferguson N.D., Caldwell E., Fan E., Camporota L., Slutsky A.S., ARDS Definition Task Force (2012). Acute Respiratory Distress Syndrome: The Berlin Definition. JAMA.

[96] Bellani G., Laffey J.G., Pham T., Fan E., Brochard L., Esteban A., Gattinoni L., van Haren F., Larsson A., McAuley D.F. (2016). Epidemiology, Patterns of Care, and Mortality for Patients with Acute Respiratory Distress Syndrome in Intensive Care Units in 50 Countries. JAMA.

[97] Matthay M.A., Arabi Y.M., Siegel E.R., Ware L.B., Bos L.D.J., Sinha P., Beitler J.R., Wick K.D., Curley M.A.Q., Constantin J.-M. (2020). Phenotypes and Personalized Medicine in the Acute Respiratory Distress Syndrome. Intensive Care Med..

[98] Sheu C.-C., Gong M.N., Zhai R., Chen F., Bajwa E.K., Clardy P.F., Gallagher D.C., Thompson B.T., Christiani D.C. (2010). Clinical Characteristics and Outcomes of Sepsis-Related vs Non-Sepsis-Related ARDS. Chest.

[99] Alhazzani W., Møller M.H., Arabi Y.M., Loeb M., Gong M.N., Fan E., Oczkowski S., Levy M.M., Derde L., Dzierba A. (2020). Surviving Sepsis Campaign: Guidelines on the Management of Critically Ill Adults with Coronavirus Disease 2019 (COVID-19). Crit. Care Med..

[100] Aziz S., Arabi Y.M., Alhazzani W., Evans L., Citerio G., Fischkoff K., Salluh J., Meyfroidt G., Alshamsi F., Oczkowski S. (2020). Managing ICU Surge during the COVID-19 Crisis: Rapid Guidelines. Intensive Care Med..

[101] Fanelli V., Ranieri V.M. (2015). Mechanisms and Clinical Consequences of Acute Lung Injury. Ann. Am. Thorac. Soc..

[102] Thompson B.T., Chambers R.C., Liu K.D. (2017). Acute Respiratory Distress Syndrome. N. Engl. J. Med..

[103] Ware L.B., Matthay M.A. (2000). The Acute Respiratory Distress Syndrome. N. Engl. J. Med..

[104] Moon H.-G., Cao Y., Yang J., Lee J.H., Choi H.S., Jin Y. (2015). Lung Epithelial Cell-Derived Extracellular Vesicles Activate Macrophage-Mediated Inflammatory Responses via ROCK1 Pathway. Cell Death Dis..

[105] Soni S., Wilson M.R., O’Dea K.P., Yoshida M., Katbeh U., Woods S.J., Takata M. (2016). Alveolar Macrophage-Derived Microvesicles Mediate Acute Lung Injury. Thorax.

[106] Lee H., Zhang D., Laskin D.L., Jin Y. (2018). Functional Evidence of Pulmonary Extracellular Vesicles in Infectious and Noninfectious Lung Inflammation. J. Immunol..

[107] Bastarache J.A., Fremont R.D., Kropski J.A., Bossert F.R., Ware L.B. (2009). Procoagulant Alveolar Microparticles in the Lungs of Patients with Acute Respiratory Distress Syndrome. Am. J. Physiol. Lung Cell. Mol. Physiol..

[108] Mahida R.Y., Price J., Lugg S.T., Li H., Parekh D., Scott A., Harrison P., Matthay M.A., Thickett D.R. (2022). CD14-Positive Extracellular Vesicles in Bronchoalveolar Lavage Fluid as a New Biomarker of Acute Respiratory Distress Syndrome. Am. J. Physiol. Lung Cell. Mol. Physiol..

[109] Papadopoulos S., Kazepidou E., Antonelou M.H., Leondaritis G., Tsapinou A., Koulouras V.P., Avgeropoulos A., Nakos G., Lekka M.E. (2020). Secretory Phospholipase A2-IIA Protein and MRNA Pools in Extracellular Vesicles of Bronchoalveolar Lavage Fluid from Patients with Early Acute Respiratory Distress Syndrome: A New Perception in the Dissemination of Inflammation?. Pharmaceuticals.

[110] Wang L., Liu J., Xie W., Li G., Yao L., Zhang R., Xu B. (2019). MiR-425 Reduction Causes Aberrant Proliferation and Collagen Synthesis through Modulating TGF-β/Smad Signaling in Acute Respiratory Distress Syndrome. Int. J. Clin. Exp. Pathol..

[111] Xu X., Liu X., Dong X., Qiu H., Yang Y., Liu L. (2022). Secretory Autophagosomes from Alveolar Macrophages Exacerbate Acute Respiratory Distress Syndrome by Releasing IL-1β. J. Inflamm. Res..

[112] Shikano S., Gon Y., Maruoka S., Shimizu T., Kozu Y., Iida Y., Hikichi M., Takahashi M., Okamoto S., Tsuya K. (2019). Increased Extracellular Vesicle MiRNA-466 Family in the Bronchoalveolar Lavage Fluid as a Precipitating Factor of ARDS. BMC Pulm. Med..

[113] Scheller N., Herold S., Kellner R., Bertrams W., Jung A.L., Janga H., Greulich T., Schulte L.N., Vogelmeier C.F., Lohmeyer J. (2019). Proviral MicroRNAs Detected in Extracellular Vesicles from Bronchoalveolar Lavage Fluid of Patients with Influenza Virus-Induced Acute Respiratory Distress Syndrome. J. Infect. Dis..

[114] Meidert A.S., Hermann S., Brandes F., Kirchner B., Buschmann D., Billaud J.-N., Klein M., Lindemann A., Aue E., Schelling G. (2021). Extracellular Vesicle Associated MiRNAs Regulate Signaling Pathways Involved in COVID-19 Pneumonia and the Progression to Severe Acute Respiratory Corona Virus-2 Syndrome. Front. Immunol..

[115] Li H., Meng X., Liang X., Gao Y., Cai S. (2015). Administration of Microparticles from Blood of the Lipopolysaccharide-Treated Rats Serves to Induce Pathologic Changes of Acute Respiratory Distress Syndrome. Exp. Biol. Med..

[116] Buesing K.L., Densmore J.C., Kaul S., Pritchard K.A., Jarzembowski J.A., Gourlay D.M., Oldham K.T. (2011). Endothelial Microparticles Induce Inflammation in Acute Lung Injury. J. Surg. Res..

[117] Densmore J.C., Signorino P.R., Ou J., Hatoum O.A., Rowe J.J., Shi Y., Kaul S., Jones D.W., Sabina R.E., Pritchard K.A. (2006). Endothelium-Derived Microparticles Induce Endothelial Dysfunction and Acute Lung Injury. Shock.

[118] Frank J.A., Briot R., Lee J.W., Ishizaka A., Uchida T., Matthay M.A. (2007). Physiological and Biochemical Markers of Alveolar Epithelial Barrier Dysfunction in Perfused Human Lungs. Am. J. Physiol. Lung Cell. Mol. Physiol..

[119] Liu A., Park J.-H., Zhang X., Sugita S., Naito Y., Lee J.-H., Kato H., Hao Q., Matthay M.A., Lee J.-W. (2019). Therapeutic Effects of Hyaluronic Acid in Bacterial Pneumonia in Ex Vivo Perfused Human Lungs. Am. J. Respir. Crit. Care Med..

[120] Morrison T.J., Jackson M.V., Cunningham E.K., Kissenpfennig A., McAuley D.F., O’Kane C.M., Krasnodembskaya A.D. (2017). Mesenchymal Stromal Cells Modulate Macrophages in Clinically Relevant Lung Injury Models by Extracellular Vesicle Mitochondrial Transfer. Am. J. Respir. Crit. Care Med..

[121] Harrington E.O., Braza J., Shil A., Chichger H. (2020). Extracellular Vesicles Released from P18 Overexpressing Pulmonary Endothelial Cells Are Barrier Protective—Potential Implications for Acute Respiratory Distress Syndrome. Pulm. Circ..

[122] Chen W., Wang S., Xiang H., Liu J., Zhang Y., Zhou S., Du T., Shan L. (2019). Microvesicles Derived from Human Wharton’s Jelly Mesenchymal Stem Cells Ameliorate Acute Lung Injury Partly Mediated by Hepatocyte Growth Factor. Int. J. Biochem. Cell Biol..

[123] Deng H., Wu L., Liu M., Zhu L., Chen Y., Zhou H., Shi X., Wei J., Zheng L., Hu X. (2020). Bone Marrow Mesenchymal Stem Cell-Derived Exosomes Attenuate LPS-Induced ARDS by Modulating Macrophage Polarization Through Inhibiting Glycolysis in Macrophages. Shock.

[124] Devaney J., Horie S., Masterson C., Elliman S., Barry F., O’Brien T., Curley G.F., O’Toole D., Laffey J.G. (2015). Human Mesenchymal Stromal Cells Decrease the Severity of Acute Lung Injury Induced by *E. coli* in the Rat. Thorax.

[125] Dutra Silva J., Su Y., Calfee C.S., Delucchi K.L., Weiss D., McAuley D.F., O’Kane C., Krasnodembskaya A.D. (2021). Mesenchymal Stromal Cell Extracellular Vesicles Rescue Mitochondrial Dysfunction and Improve Barrier Integrity in Clinically Relevant Models of ARDS. Eur. Respir. J..

[126] Hayes M., Curley G.F., Masterson C., Devaney J., O’Toole D., Laffey J.G. (2015). Mesenchymal Stromal Cells Are More Effective than the MSC Secretome in Diminishing Injury and Enhancing Recovery Following Ventilator-Induced Lung Injury. Intensive Care Med. Exp..

[127] Huang R., Qin C., Wang J., Hu Y., Zheng G., Qiu G., Ge M., Tao H., Shu Q., Xu J. (2019). Differential Effects of Extracellular Vesicles from Aging and Young Mesenchymal Stem Cells in Acute Lung Injury. Aging.

[128] Ionescu L., Byrne R.N., van Haaften T., Vadivel A., Alphonse R.S., Rey-Parra G.J., Weissmann G., Hall A., Eaton F., Thébaud B. (2012). Stem Cell Conditioned Medium Improves Acute Lung Injury in Mice: In Vivo Evidence for Stem Cell Paracrine Action. Am. J. Physiol. Lung Cell. Mol. Physiol..

[129] Kaspi H., Semo J., Abramov N., Dekel C., Lindborg S., Kern R., Lebovits C., Aricha R. (2021). MSC-NTF (NurOwn^®^) Exosomes: A Novel Therapeutic Modality in the Mouse LPS-Induced ARDS Model. Stem Cell Res. Ther..

[130] Mao G.-C., Gong C.-C., Wang Z., Sun M.-X., Pei Z.-P., Meng W.-Q., Cen J.-F., He X.-W., Lu Y., Xu Q.-Q. (2021). BMSC-Derived Exosomes Ameliorate Sulfur Mustard-Induced Acute Lung Injury by Regulating the GPRC5A-YAP Axis. Acta Pharmacol. Sin..

[131] Monsel A., Zhu Y., Gennai S., Hao Q., Hu S., Rouby J.-J., Rosenzwajg M., Matthay M.A., Lee J.W. (2015). Therapeutic Effects of Human Mesenchymal Stem Cell-Derived Microvesicles in Severe Pneumonia in Mice. Am. J. Respir. Crit. Care Med..

[132] Silva J.D., de Castro L.L., Braga C.L., Oliveira G.P., Trivelin S.A., Barbosa-Junior C.M., Morales M.M., Dos Santos C.C., Weiss D.J., Lopes-Pacheco M. (2019). Mesenchymal Stromal Cells Are More Effective Than Their Extracellular Vesicles at Reducing Lung Injury Regardless of Acute Respiratory Distress Syndrome Etiology. Stem Cells Int..

[133] Su V.Y.-F., Lin C.-S., Hung S.-C., Yang K.-Y. (2019). Mesenchymal Stem Cell-Conditioned Medium Induces Neutrophil Apoptosis Associated with Inhibition of the NF-ΚB Pathway in Endotoxin-Induced Acute Lung Injury. Int. J. Mol. Sci..

[134] Tang X.-D., Shi L., Monsel A., Li X.-Y., Zhu H.-L., Zhu Y.-G., Qu J.-M. (2017). Mesenchymal Stem Cell Microvesicles Attenuate Acute Lung Injury in Mice Partly Mediated by Ang-1 MRNA. Stem Cells.

[135] Varkouhi A.K., Jerkic M., Ormesher L., Gagnon S., Goyal S., Rabani R., Masterson C., Spring C., Chen P.Z., Gu F.X. (2019). Extracellular Vesicles from Interferon-γ-Primed Human Umbilical Cord Mesenchymal Stromal Cells Reduce *Escherichia coli*-Induced Acute Lung Injury in Rats. Anesthesiology.

[136] Wei X., Yi X., Lv H., Sui X., Lu P., Li L., An Y., Yang Y., Yi H., Chen G. (2020). MicroRNA-377-3p Released by Mesenchymal Stem Cell Exosomes Ameliorates Lipopolysaccharide-Induced Acute Lung Injury by Targeting RPTOR to Induce Autophagy. Cell Death Dis..

[137] Wu X., Liu Z., Hu L., Gu W., Zhu L. (2018). Exosomes Derived from Endothelial Progenitor Cells Ameliorate Acute Lung Injury by Transferring MiR-126. Exp. Cell Res..

[138] Zhu Y.-G., Feng X.-M., Abbott J., Fang X.-H., Hao Q., Monsel A., Qu J.-M., Matthay M.A., Lee J.W. (2014). Human Mesenchymal Stem Cell Microvesicles for Treatment of *Escherichia coli* Endotoxin-Induced Acute Lung Injury in Mice. Stem Cells.

[139] Shah T., Qin S., Vashi M., Predescu D.N., Jeganathan N., Bardita C., Ganesh B., diBartolo S., Fogg L.F., Balk R.A. (2018). Alk5/Runx1 Signaling Mediated by Extracellular Vesicles Promotes Vascular Repair in Acute Respiratory Distress Syndrome. Clin. Transl. Med..

[140] Guervilly C., Lacroix R., Forel J.-M., Roch A., Camoin-Jau L., Papazian L., Dignat-George F. (2011). High Levels of Circulating Leukocyte Microparticles Are Associated with Better Outcome in Acute Respiratory Distress Syndrome. Crit. Care.

[141] Shaver C.M., Woods J., Clune J.K., Grove B.S., Wickersham N.E., McNeil J.B., Shemancik G., Ware L.B., Bastarache J.A. (2017). Circulating Microparticle Levels Are Reduced in Patients with ARDS. Crit. Care.

[142] Park J., Kim S., Lim H., Liu A., Hu S., Lee J., Zhuo H., Hao Q., Matthay M.A., Lee J.W. (2019). Therapeutic Effects of Human Mesenchymal Stem Cell Microvesicles in an Ex Vivo Perfused Human Lung Injured with Severe *E. coli* Pneumonia. Thorax.

[143] Sengupta V., Sengupta S., Lazo A., Woods P., Nolan A., Bremer N. (2020). Exosomes Derived from Bone Marrow Mesenchymal Stem Cells as Treatment for Severe COVID-19. Stem Cells Dev..

[144] Dilogo I.H., Aditianingsih D., Sugiarto A., Burhan E., Damayanti T., Sitompul P.A., Mariana N., Antarianto R.D., Liem I.K., Kispa T. (2021). Umbilical Cord Mesenchymal Stromal Cells as Critical COVID-19 Adjuvant Therapy: A Randomized Controlled Trial. Stem Cells Transl. Med..

[145] Lanzoni G., Linetsky E., Correa D., Messinger Cayetano S., Alvarez R.A., Kouroupis D., Alvarez Gil A., Poggioli R., Ruiz P., Marttos A.C. (2021). Umbilical Cord Mesenchymal Stem Cells for COVID-19 Acute Respiratory Distress Syndrome: A Double-blind, Phase 1/2a, Randomized Controlled Trial. Stem Cells Transl. Med..

[146] Zheng G., Huang L., Tong H., Shu Q., Hu Y., Ge M., Deng K., Zhang L., Zou B., Cheng B. (2014). Treatment of Acute Respiratory Distress Syndrome with Allogeneic Adipose-Derived Mesenchymal Stem Cells: A Randomized, Placebo-Controlled Pilot Study. Respir. Res..

[147] Yip H.-K., Fang W.-F., Li Y.-C., Lee F.-Y., Lee C.-H., Pei S.-N., Ma M.-C., Chen K.-H., Sung P.-H., Lee M.S. (2020). Human Umbilical Cord-Derived Mesenchymal Stem Cells for Acute Respiratory Distress Syndrome. Crit. Care Med..

[148] Chen J., Hu C., Chen L., Tang L., Zhu Y., Xu X., Chen L., Gao H., Lu X., Yu L. (2020). Clinical Study of Mesenchymal Stem Cell Treatment for Acute Respiratory Distress Syndrome Induced by Epidemic Influenza A (H7N9) Infection: A Hint for COVID-19 Treatment. Engineering.

[149] Bellingan G., Jacono F., Bannard-Smith J., Brealey D., Meyer N., Thickett D., Young D., Bentley A., McVerry B.J., Wunderink R.G. (2021). Safety and Efficacy of Multipotent Adult Progenitor Cells in Acute Respiratory Distress Syndrome (MUST-ARDS): A Multicentre, Randomised, Double-Blind, Placebo-Controlled Phase 1/2 Trial. Intensive Care Med..

[150] Husain-Syed F., Slutsky A.S., Ronco C. (2016). Lung-Kidney Cross-Talk in the Critically Ill Patient. Am. J. Respir. Crit. Care Med..

[151] Mahida R.Y., Matsumoto S., Matthay M.A. (2020). Extracellular Vesicles: A New Frontier for Research in Acute Respiratory Distress Syndrome. Am. J. Respir. Cell Mol. Biol..

[152] Song Y., Dou H., Li X., Zhao X., Li Y., Liu D., Ji J., Liu F., Ding L., Ni Y. (2017). Exosomal MiR-146a Contributes to the Enhanced Therapeutic Efficacy of Interleukin-1β-Primed Mesenchymal Stem Cells Against Sepsis. Stem Cells.

[153] Ju Z., Ma J., Wang C., Yu J., Qiao Y., Hei F. (2017). Exosomes from IPSCs Delivering SiRNA Attenuate Intracellular Adhesion Molecule-1 Expression and Neutrophils Adhesion in Pulmonary Microvascular Endothelial Cells. Inflammation.

[154] Aghajani Nargesi A., Lerman L.O., Eirin A. (2017). Mesenchymal Stem Cell-Derived Extracellular Vesicles for Kidney Repair: Current Status and Looming Challenges. Stem Cell Res. Ther..

